# Comparative Analysis of Host-Associated Variation in *Phytophthora cactorum*

**DOI:** 10.3389/fmicb.2021.679936

**Published:** 2021-07-02

**Authors:** Charlotte F. Nellist, Andrew D. Armitage, Helen J. Bates, Maria K. Sobczyk, Matteo Luberti, Laura A. Lewis, Richard J. Harrison

**Affiliations:** ^1^NIAB EMR, East Malling, United Kingdom; ^2^NIAB, Cambridge, United Kingdom; ^3^National Resources Institute, University of Greenwich, Chatham, United Kingdom

**Keywords:** crown rot, oomycete, phylogenomics, effectors, cryptic species

## Abstract

*Phytophthora cactorum* is often described as a generalist pathogen, with isolates causing disease in a range of plant species. It is the causative agent of two diseases in the cultivated strawberry, crown rot (CR; causing whole plant collapse) and leather rot (LR; affecting the fruit). In the cultivated apple, *P. cactorum* causes girdling bark rots on the scion (collar rot) and rootstock (crown rot), as well as necrosis of the fine root system (root rot) and fruit rots. We investigated evidence for host specialisation within *P. cactorum* through comparative genomic analysis of 18 isolates. Whole genome phylogenetic analysis provided genomic support for discrete lineages within *P. cactorum*, with well-supported non-recombining clades for strawberry CR and apple infecting isolates specialised to strawberry crowns and apple tissue. Isolates of strawberry CR are genetically similar globally, while there is more diversity in apple-infecting isolates. We sought to identify the genetic basis of host specialisation, demonstrating gain and loss of effector complements within the *P. cactorum* phylogeny, representing putative determinants of host boundaries. Transcriptomic analysis highlighted that those effectors found to be specific to a single host or expanded in the strawberry lineage are amongst those most highly expressed during infection of strawberry and give a wider insight into the key effectors active during strawberry infection. Many effectors that had homologues in other *Phytophthoras* that have been characterised as avirulence genes were present but not expressed in our tested isolate. Our results highlight several RxLR-containing effectors that warrant further investigation to determine whether they are indeed virulence factors and host-specificity determinants for strawberry and apple. Furthermore, additional work is required to determine whether these effectors are suitable targets to focus attention on for future resistance breeding efforts.

## Introduction

The *Phytophthora* genus serves as a model system for studying evolution of pathogenicity and resistance in plant pathosystems. Over 150 species have been named in the genus, with many pathogenic on plants (Yang et al., 2017). *Phytophthora* spp. are extremely effective plant pathogens that are able to disperse and infect hosts via the asexual, motile stage of their life cycle, zoospores, as well as able to resist and survive for many years in unfavourable conditions as thick-walled sexual oospores. Many *Phytophthora* spp. are specialised and only able to colonise one or a few host plants, for example *Phytophthora fragariae* which is thought to only colonise strawberry. Some species, such as *Phytophthora cactorum* (Lebert and Cohn) Schroet, are traditionally considered to be generalists and are able to cause disease on a broad range of plant species, including herbaceous and woody plants (Erwin and Ribeiro, 1996). Two examples of these hosts in the *Rosaceae* family, are the herbaceous cultivated strawberry (*Fragaria* × *ananassa*) and woody cultivated apple (*Malus* × *domestica*).

*Phytophthora cactorum* is the causative agent of two diseases in the cultivated strawberry, crown rot (CR; causing whole plant collapse (Deutschmann, 1954); and leather rot (LR; affecting the fruit (Rose, 1924). Both are considered major diseases of strawberry in temperate regions, with crop losses of up to 40 and 30% reported, respectively, for each disease (Ellis and Grove, 1983; Stensvand et al., 1999). The majority of commercial strawberries grown in the UK are grown under polytunnels or in glasshouses, on tabletops using soilless substrate, such as coir (coconut husk) (Boyer et al., 2016). *P. cactorum* is a particular problem in this production system due to ease of spread through the irrigation system via the motile asexual life-stage of *Phytophthora*, zoospores. Introduction into the irrigation system through asymptomatic infections in planting material represents the biggest risk to growers. Nursery propagation of strawberry is still based in the field, where the presence of resident pathogen inoculum in the soil along with latent infection in plants represent a severe threat to production. A study in 2018 of UK strawberry planting material (runners), commissioned by the Agriculture and Horticulture Development Board (AHDB), reported incidences of *P. cactorum* as high as 30% and great variation observed between batches of plants tested, but on average an incidence of 8–10% was recorded (Wedgwood et al., 2020).

In the cultivated apple, *P. cactorum* causes girdling bark rots on the scion (collar rot) and rootstock (crown rot), as well as necrosis of the fine root system (root rot) and fruit rots (Harris, 1991). *P. cactorum* affects nearly all apple growing regions of the world, debilitating the trees and leading to reduced fruit yield and eventual tree death (Alexander and Stewart, 2001). Due to the high costs associated with orchard establishment, losses due to *P. cactorum* can result in significant economic losses. As *P. cactorum* is homothallic (self-fertile), it produces sexual oospores which can survive for long periods in the soil, growing material and plant material, contributing to its importance as a worldwide threat to apple production.

*Phytophthora cactorum* diverged from other Clade 1 *Phytophthora* spp., *P. infestans* and *P. parasitica*, an estimated 221.4 Ma (138.6–342.4 million years ago; Yang et al., 2018) which is some 100 My (million years) earlier than the divergence of the Dryadoideae, Ammygdaloieae, and Rosioideae and some 150–170 My before the emergence of the Fragariae (Zhang et al., 2017). *P. cactorum* specifically belongs to subclade 1a, along with *P. idaei* (Yang et al., 2017), a sister taxon and pathogen of another important *Rosaceae* crop, raspberry (*Rubus idaeus*). Despite such a broad host range being described for *P. cactorum*, host specialisation has been documented to particular plant species, including strawberry and apple (Seemüller and Schmidle, 1979). *P. cactorum* isolates originating from strawberry crowns were found to be less pathogenic on apple bark tissue than isolates originating from strawberry fruit or apple and vice versa, with apple and strawberry fruit isolates being less pathogenic on strawberry crowns. It was found that all pathotypes were able to cause disease in strawberry fruit (Seemüller and Schmidle, 1979). *P. cactorum* host specialisation has also been reported in other hosts, such as silver birch, peach and almond (Hantula et al., 1997, 2000; Lilja et al., 1998; Thomidis, 2003; Bhat et al., 2006). The specific genetic components responsible for host specialisation in *P. cactorum* are not known. Although, studies in both filamentous and bacterial pathogen systems support the model of effector repertoire being one of the key determinants in pathogen host range and non-host resistance (Schulze-Lefert and Panstruga, 2011; Stam et al., 2014).

Genomic resources have recently become available for *P. cactorum*, with genomes released for isolates from beech, *Fagus sylvatica* (Grenville-Briggs et al., 2017), Chinese ginseng, *Panax notoginseng* (Yang et al., 2018) and strawberry, *F. x ananassa* (Armitage et al., 2018). *Phytophthora* spp. carry two major families of cytoplasmic effectors that are translocated into the host cell, RxLR and Crinklers, both have been characterised by specific motifs and consist of a rapidly evolving effector/modulating domain. The RxLR family of effectors are characterised by an N-terminal signal peptide, followed by an RxLR-s/dEER motif (Arginine, any amino-acid, Leucine, Arginine often followed by Serine/Aspartate, Glutamate, Glutamate, Arginine) and a variable C-terminal domain that may contain WY domain repeats (Wawra et al., 2012; Win et al., 2012). These effectors are renowned for suppressing host defence mechanisms through the manipulation of various aspects of plant defence (Anderson et al., 2015). The second family of cytoplasmic effectors are Crinklers (CRNs), named because of the leaf crinkling effect observed when expressed in host plants (Torto et al., 2003). They are characterised by an N-terminal LFLAK domain followed by a DWL domain, with a DI domain sometimes present between these two domains (Haas et al., 2009; Stam et al., 2013). These conserved domains are followed by variable C-terminal domains.

In addition to cytoplasmic effectors, *Phytophthora* spp. also deploy an arsenal of apoplastic effector proteins during infection, including a large number of hydrolytic enzymes, such as cutinases, glycoside hydrolases (GHs), pectinases, and proteases, which promote their infection and manipulation of the plant immune system (Armitage et al., 2018; Wang and Wang, 2018a,b). They also encode members of extracellular phytotoxin families such as the conserved necrosis-inducing proteins (NLPs) and small cysteine-rich (SCR) proteins, for such as PcF (*P. cactorum* factor) (Orsomando et al., 2001) and INF1 (Kamoun et al., 1997).

Here, we further explore host specialisation and the basis of pathogenicity in *P. cactorum* by investigating multiple isolates collected from symptomatic strawberry crowns and fruit, as well as isolates from symptomatic apple tissue. We demonstrate that there are distinct lineages within *P. cactorum* showing adaptation to strawberry crowns and apple tissue. We show that these lineages are associated with unique effector complements and that these differential genes are highly expressed during plant infection. Taken together, this work elucidates key lineage specific effector genes playing roles in specialisation to strawberry and apple in *P. cactorum*.

## Materials and Methods

### *Phytophthora* Isolates Investigated in This Study

Eighteen *P. cactorum* isolates and three *P. idaei* isolates (detailed in Table 1) were investigated in this study. Of the 18 *P. cactorum* isolates, 13 were isolated from strawberry crown tissue exhibiting crown rot symptoms, two from strawberry fruit exhibiting leather rot symptoms and three isolated from symptomatic apple bark. One of the crown rot isolates, 10300, has been previously published by Armitage et al. (2018). The three isolates of *P. idaei* were isolated from infected raspberry material. All isolates were revived and maintained on V8 agar at 20°C in the dark.

**Table 1:** Summary of *Phytophthora cactorum* and *Phytophthora idaei* isolates used in this study.

**Isolate ID**	**Genome ID**	**GenBank accession**	**Species**	**Material isolated from**	**Year**	**Location**	**Previously published**
P414	Pcac1	NHQK00000000	*P. cactorum*	Strawberry crown	2011	Somerset, UK	
P404	PC116	RCMJ00000000	*P. cactorum*	Strawberry crown	1998	UK	
P415	PC118	RCML00000000	*P. cactorum*	Strawberry crown	2013	UK	
P416	PC119	RCMM00000000	*P. cactorum*	Strawberry crown		UK	
P421	PC129	RCMV00000000	*P. cactorum*	Strawberry crown	2017	UK	
PC13/15	PC122	RCMP00000000	*P. cactorum*	Strawberry crown	2015	UK	
10300	PC110	GCA_003287315.1	*P. cactorum*	Strawberry crown	2006	Norway	Armitage et al., 2018
4032	PC115	RCMI00000000	*P. cactorum*	Strawberry crown		Netherlands	
4040	PC117	RCMK00000000	*P. cactorum*	Strawberry crown		Netherlands	
2003-3	PC114	RCMH00000000	*P. cactorum*	Strawberry crown		Netherlands	
12–420	PC111	RCME00000000	*P. cactorum*	Strawberry crown	2012	Florida, USA	
15–7	PC113	RCMG00000000	*P. cactorum*	Strawberry crown	2015	Florida, USA	
15–13	PC112	RCMF00000000	*P. cactorum*	Strawberry crown	2015	Florida, USA	
11–40	PC127	RCMT00000000	*P. cactorum*	Strawberry fruit	2011	Florida, USA	
17–21	PC128	RCMU00000000	*P. cactorum*	Strawberry fruit	2017	Florida, USA	
62471	PC120	RCMN00000000	*P. cactorum*	Apple	2014	Kent, UK	
P295	PC121	RCMO00000000	*P. cactorum*	Apple (collar rot)	1984	Offham, UK	
R36/14	PC123	RCMQ00000000	*P. cactorum*	Apple	2014	Kent, UK	
LV007^a^		GCA_002081965.1	*P. cactorum*	European Beech	2016	Sweden	Grenville-Briggs et al., 2017
SCRP370	PI125	RCMR00000000	*P. idaei*	Raspberry	1985	Scotland, UK	
SCRP371	PI124	QOKR00000000	*P. idaei*	Raspberry	1986	England, UK	
SCRP376	PI126	RCMS00000000	*P. idaei*	Raspberry	1993	England, UK	

a *Only sequence data was used in this study*.

### Whole Genome Sequencing and Assembly

Mycelia of *P. cactorum* and *P. idaei* were grown in clarified V8-juice broth, similar to Wilcox et al. (1993); comprised of 100 mL V8-juice (Arnotts Biscuits Limited), 1.4 g calcium carbonate (CaCO_3_; Sigma Aldrich) and 100 mL dH_2_O, which were centrifuged at 2,500 × *g* for 15 min, the supernatant was decanted and made up to 1,600 mL with dH_2_O; 200 mL aliquots were dispensed into 250 mL flasks and autoclaved for 20 min at 120°C. Five mycelial plugs per isolate were added to each flask and were grown at 20°C under lab light/dark cycle for 10 days in a shaker incubator set to 200 rpm (revolutions per minute). The mycelia were washed in sterile dH_2_O, vacuum filtered and freeze dried overnight.

The GenElute Plant Genomic DNA Kit (Sigma) was used to extract gDNA from *P. idaei* isolate, SCRP371. gDNA was sonicated in a water bath and size selected, ~500 bp, on an agarose gel and extracted. An Illumina library was constructed using the TruSeq LT Kit (FC-121-2001) and was sequenced using Illumina MiSeq v3 2x 300 bp PE Reagent Kit. For all remaining isolates gDNA was extracted using the Macherey-Nagel Nucleospin Plant II Kit (Fisher Scientific). gDNA was sheared using the Covaris M220 with microTUBE-50 (Covaris) and size selected, 450−600 bp, using a Blue Pippin (Sage Science). Illumina libraries were constructed using a PCR-free method using NEBNext End Repair (E6050S), NEBNext dA-tailing (E6053S) and Blunt T/A ligase (M0367S) New England Biolabs modules. Library insert sizes were 400–600 bp and were sequenced using Illumina Miseq v2 2x 250 bp paired-end (PE; MS-102-2003) or v3 2x 300 bp PE (MS-102-3003) Reagent Kits.

A single *P. cactorum* isolate (P414), from a symptomatic crown of strawberry, was selected for additional PacBio sequencing. gDNA extraction was performed using the Genomic-tip DNA 100/G Kit (Qiagen), following the Tissue Sample method. A minimum of 20 μg of gDNA at approximately 100 ng/μL concentration, with a 260/280 ratio of 1.88 and a 260/230 ratio of 2.26, and a minimum molecular weight of 40 kb was sent to The Earlham Institute, UK. The large insert library was prepared by The Earlham Institute according to manufacturer specifications and sequenced to achieve approximately 87 times coverage on a PacBio RSII platform, using P6-C4 chemistry.

A long-read *de novo* assembly was generated for isolate P414 by first performing read correction and trimming using Canu v1.6 (Koren et al., 2017), before assembling with SMARTdenovo (February 26, 2017 github commit). Errors in this SMARTdenovo assembly were polished through five iterations of Pilon v1.17 (Walker et al., 2014), using the “diploid” flag and trimmed Illumina reads. Illumina reads were trimmed to remove low quality bases and Illumina adapters with fastq-mcf v1.04.676 (Aronesty, 2013).

*De novo* assembly of MiSeq data for the remaining 19 genomes was performed using SPAdes v.3.11.0 (Bankevich et al., 2012). Assembly statistics were collected for all assemblies using QUAST v3.0 (Gurevich et al., 2013). Completeness of the *Phytophthora* genome assemblies was assessed by analysis of conserved Benchmarking Universal Single-Copy Orthologue (BUSCO, v3; Simão et al., 2015; Waterhouse et al., 2017) genes using the Alveolata-Stramenopiles dataset. DeconSeq was run on all assemblies to remove any potential bacterial contaminants with homology to databases of all “complete” *Bacillus* or *Paenobacillus* genomes as downloaded from NCBI (Schmieder and Edwards, 2011). A database of *Phytophthora* contigs was also made and contigs that showed homology to both bacterial and *Phytophthora* databases were retained. Assemblies were edited in accordance with results from the NCBI contamination screen (run as part of submission to GenBank in December 2017) with contigs split, trimmed or excluded as required. RepeatModeler, RepeatMasker and transposonPSI were used to identify repetitive and low complexity regions (http://www.repeatmasker.org, http://transposonpsi.sourceforge.net).

### Gene and Open Reading Frame Prediction and Functional Annotation

Gene prediction was performed following Armitage et al. (2018) and detailed is in Supplementary Materials and Methods. Gene models were also augmented with further effector candidates from open reading frames (ORFs) using the methods previously described in Armitage et al. (2018).

Functional annotation of gene models was performed as described previously in Armitage et al. (2018), further details can be found in Supplementary Materials and Methods. A publicly available *P. cactorum* genome, LV007, isolated from European Beech (*Fagus sylvatica*) was downloaded from GenBank (PRJNA380728; Grenville-Briggs et al., 2017) and used in the subsequent analyses (detailed in Table 1).

### Phylogenetics

A phylogeny was determined from conserved single copy genes present in *P. cactorum* genomes and in *P. idaei* outgroup isolates. Partial and complete single hits from BUSCO searches, using the Alveolata-Stramenopiles obd9 database, were extracted from the 20 sequenced genomes, as well as the publicly available *P. cactorum* 10300 (Armitage et al., 2018) and LV007 genomes (Grenville-Briggs et al., 2017). This led to retention of nucleotide sequences for 230/234 loci, which were aligned using MAFFT v6.864b (Katoh and Standley, 2013), before being trimmed with trimAl v.1.4.1 (Capella-Gutiérrez et al., 2009). A maximum likelihood tree was determined for each locus using RAxML v.8.1.17 (Liu et al., 2011), with the most parsimonious tree for each locus used to determine an overall consensus phylogeny across all 230 loci using ASTRAL v.5.6.1 (Zhang et al., 2018). The resulting tree was visualised using the R package GGtree v.1.12.4 (Yu et al., 2016).

### SNP and Variant Calling

Single Nucleotide Polymorphisms (SNPs), indels and structural variants were identified in reference to the *P. cactorum* P414 genome. Trimmed Illumina reads from each isolate were aligned to the P414 genome using Bowtie2 v2.2.6 (Langmead and Salzberg, 2012), with SNP variants identified using GATK (McKenna et al., 2010; DePristo et al., 2011; Auwera et al., 2013) and indels/structural variants identified using SvABA (Wala et al., 2018). SNPs called by GATK were filtered using VCFtools (Danecek et al., 2011), retaining bi-allelic SNPs with an QUAL > 30, MQ > 40, DP > 10, GQ > 30. SNP calls were also filtered if isolate P414 Illumina reads showed a homozygous polymorphism in reference to the P414 assembly as these represent errors in the assembly rather than SNP variants. Effects of predicted variants on *P. cactorum* gene models were established using SnpEff (Cingolani et al., 2012). Population genetic statistics were calculated from SNP variants using VCFtools and the R package PopGenome (Pfeifer et al., 2014). Structure analysis was performed using FastSTRUCTURE v1.0 (Raj et al., 2014). The program was run with *k* values between 1 and 6 and number of populations determined where *k* maximised marginal likelihood. DISTRUCT plots were generated from output meanQ files using R-studio v1.1.453. A SNP distance matrix was made showing the number of variants that differ between isolates. SNP variants were extracted from the final.vcf file as a fasta alignment of concatenated variable sites, containing two sequences per isolate (representing the first and second allele called at each site, respectively). A distance matrix was calculated in Geneious Prime v2020.0 and exported into Microsoft Excel.

### *Phytophthora* spp. Zoospore Production

The production of zoospores was followed from Nellist et al. (2019) and is detailed in the Supplementary Materials and Methods. The concentration of zoospores was determined using a haemocytometer and adjusted to 1 × 10^4^, 2 × 10^4^ or 5 × 10^3^ zoospores per mL by diluting with dilute compost extract. The adjusted solution was kept on ice until ready to be used to inoculate plants/unripe fruit.

### Pathogenicity Tests on Strawberry Crowns

The virulence of the 18 *P. cactorum* and three *P. ideai* isolates were tested on the crowns of 10 clonal replicates of three cultivars of cultivated strawberry (*F*. × *ananassa*). ‘Malling Opal,' an extremely crown rot susceptible cultivar, ‘Elsanta' a susceptible cultivar and ‘Fenella' a cultivar with good resistance to *P. cactorum*, were screened.

The preparation of plant material is detailed in Supplementary Materials and Methods and the inoculation procedure for coldstored strawberry plants was performed as described in Nellist et al. (2019). The data for the ten replicates were averaged and a mean crown rot disease score was used for further analysis. Statistical analyses were performed using R (v3.6.0, “Planting of a Tree”; R Core Team, 2019). A one-way ANOVA was performed to analyse the difference between the pathogenicity of isolates cultured from strawberry on the three cultivars of strawberry.

### Pathogenicity Tests on Detached Unripe Strawberry Fruit

Unripe ‘Elsanta' strawberry fruit were picked while still white/green in the Summer of 2018. The fruit were then surface sterilised by immersion in a 10% bleach solution and then rinsed twice with dH_2_O. The fruit were dried off and two fruit were placed into each sterile 90 mm triple-vented petri dish bottom or lid (Thermo Scientific). The petri dish lids and bottoms with fruit were placed on trays sterilised with 70% ethanol. A sterile 4 mm cork borer was used to bore a shallow hole in the fruit. Zoospores were produced as described above and 100 μL of 5 × 10^3^ zoospore suspension was added into the hole of the fruit. Fruit were screened in three separate experiments with a minimum of eight replicates per isolate screened in experiment. The trays were then sealed in a plastic bag and left in the dark at 20°C. The ratios of colonised to non-colonised fruit were recorded after 7 days.

### Pathogenicity Tests on Excised Apple Shoots

Dormant first year growth apple shoots were collected from ‘Cox' and ‘Gala' in the Winter 2018. The processing of apple shoots was followed from Luberti et al. (2021) and is detailed in the Supplementary Materials and Methods. Shoots were assessed for maximum lesion length at 4 weeks by removing the bark around the wound using a scalpel. A digital calliper was used to take measurements and the original wound size, 4 mm, was subtracted from each measurement. A one-way ANOVA was performed to analyse the difference between the pathogenicity of isolates cultured from apple and strawberry fruit on the two cultivars of apple.

### *In vitro* Strawberry Root Pathogenicity Transcriptome Analysis

Transcriptome changes during host infection were investigated through an infection time-course on strawberry roots infected with *P. cactorum* isolate P414. The time-course was performed in the susceptible cultivar ‘Emily' and moderately resistant cultivar ‘Fenella'; parents of a mapping population used in a previous study (Nellist et al., 2019). Micropropagated plants were produced by GenTech Propagation Ltd. for these experiments. Upon arrival at NIAB EMR, plants were transferred to 120 × 120 × 15 mm, four vent, petri dishes (Corning, Gosselin), half filled with ATS (*Arabidopsis thaliana* salts) media, two plants per plate. ATS media was prepared as described by Taylor et al. (2016). The media were poured into the bottom plate and after it had set, half of the agar was excised with a sterile flat spatula. Plants were then transplanted so the crown sat on the top of the agar (Supplementary Figure 1A). The roots were gently smoothed down, ensuring they were touching the agar. The plates were then sealed with Sellotape and aluminium foil cases were made to surround the agar ensuring a dark environment for the root system (Supplementary Figure 1B). The plates were then positioned upright in a growth cabinet (Panasonic MLR-325H) at 22°C, on a 16/8 h, day/night light cycle with a photosynthetic photon flux (PPF) of 150 μmol m^−2^ s^−1^ provided by fluorescent lamps (FL40SSENW37).

Just before inoculation, the micropropagated ‘Emily' and ‘Fenella' plants were transferred to fresh plates and zoospores were produced as described above. Each root system was inoculated with 1 mL of 2 × 10^4^ zoospore suspension, using a pipette and slowly dripping the suspension over the entire root system. The plates were then sealed with Sellotape, partially recovered with the aluminium foil and were kept flat for 2 h to allow the zoospores to encyst. Mock inoculated (0 h post inoculation) were inoculated with 1 mL of dilute compost extract. The plates were then returned to their upright position until harvested. Root samples were collected at 0 (mock), 6, 12, 24, 48, 72, 96, 120, and 144 h post inoculation (hpi). The root systems were swilled in sterile dH_2_O to remove any agar, patted dry and were collected in 2 mL Eppendorf microcentrifuge tubes, flash frozen in liquid nitrogen and stored at −80°C.

Total RNA was extracted from the strawberry roots following a modified version of Yu et al. (2012), over 2 days, detailed in Supplementary Materials and Methods. At least 1 μg of root RNA with a RIN score above 7 and with 260/280 and 260/230 ratios above 1.8 were sent to Novogene for sequencing. Strawberry root samples were sequenced to a depth of 50 million reads per sample.

Timepoints for sequencing were selected through the detection of β-tubulin transcripts by Reverse Transcriptase-PCR, using SuperScript^TM^ III Reverse Transcriptase kit with an equal amount of RNA used for each sample. The complementary DNA (cDNA) was then analysed by PCR with 200 μM dNTPs, 0.2 μM of each primer (detailed in Supplementary Table 1), 2 μL of cDNA template and 2.5 units of Taq DNA polymerase and the buffer supplied in a 20 μL reaction. Reactions were conducted in a Veriti 96-well thermocycler with an initial denaturation step at 95°C for 30 s, followed by 35 cycles of a denaturation step at 95°C for 30 s, an annealing temperature of 60°C for 30 s and an extension step of 72°C for 30 s. This was followed by a final extension step of 72°C for 5 min and held at 10°C. Products were visualised by gel electrophoresis on a 1% w/v agarose gel at 80 V for 90 min, stained with GelRed. Following this, three biological replicates of samples taken at: 0, 12 and 48 hpi for both ‘Emily' and ‘Fenella' were sequenced (Supplementary Figure 3).

Mycelia of P414 were grown in clarified V8-juice broth as described as above with the addition of 500 μg/mL of ampicillin (Fisher) and 10 μg/mL of rifampicin (Fisher) at 20°C under lab light/dark cycle for 10 days in a shaker incubator set to 200 rpm. The mycelia were washed in sterile dH_2_O, vacuum filtered, flash frozen in liquid nitrogen and stored at −80°C. Total RNA was extracted from three biological replicates of flash frozen P414 mycelia using the RNeasy Mini Kit (Qiagen) following the manufacturer's protocol. RNA quality and quantity were assessed as described above and the RNA was sent to The Earlham Institute, UK for sequencing. cDNA library insert sizes were 450–625 bp and were sequenced on Illumina HiSeq4000, using 2x 150 bp PE Reagent Kit. Three barcoded biological replicates of each treatment were pooled and sequenced across multiple lanes. *P. cactorum* mycelia were sequenced to a depth of 25 million reads per sample.

Illumina adapters and low-quality bases were trimmed using fastq-mcf. All RNAseq data were aligned to the *Fragaria vesca* genome v1.1 (Shulaev et al., 2011) using STAR v2.5.3a (Dobin et al., 2013), to remove strawberry reads from the dataset. Read alignment for differential gene expression was performed using Salmon v0.9.1 (Patro et al., 2017) with differential gene expression during infection investigated using DESeq2 (Love et al., 2014). The normalised expression value was represented by applying Fragments per Kilobase of exon model per Million mapped reads (FPKM). All mycelial genes with an FPKM value <1 were adjusted to 1. The Log Fold Change (LFC) in gene expression was calculated using adjusted FPKM values to prevent overprediction of LFC from low-expressed/unexpressed genes under one condition, with all genes with an FPKM value <1 were adjusted 1; LFC = log_2_(‘Emily' 12 hpi FPKM)/log_2_(Adjusted Mycelium FPKM). Genes were designated as differentially expressed if they had a DeSeq2 P-adj <0.05 and LFC was ≥2 or was ≤ −2. The top 100 expressed genes were investigated further, based on LFC in descending order of ‘Emily' at 12 hpi vs. Mycelium. Temporal expression was assessed by identifying those differentially expressed genes with a consistent in peak expression (based upon LFC) in both ‘Emily' and ‘Fenella' at 12 hpi (early-expressed genes) or 48 hpi (late-expressed genes).

### Comparative Genomics of Known Virulence Related Factors

A selection of known RxLR *Avr* gene sequences (amino acid sequence after the signal peptide) were BLASTed against the 22 *Phytophthora* genomes in Geneious Prime v2020.0 and the hits (tBLASTx *E* > 1 × 10^−10^) were investigated. Expression of interesting hits were analysed using the *in planta* RNAseq data and interesting candidates were further explored in the representative isolates in the unripe fruit assay.

### Strawberry Fruit Reverse Transcription Quantitative Polymerase Chain Reaction Screen

Unripe, green/white “Driscoll® Amesti™” strawberry fruit were used for the pathogenicity time course of three *P. cactorum* isolates; P414, R36/14, and 17-21. The fruit were sterilised and prepared as described above. Zoospores were produced as described above and 100 μL of 5 × 10^3^ zoospore suspension was added into the hole of each fruit. Samples were taken at 0, 36, 48, and 60 hpi. A larger cork borer of 10 mm was used to excise an area around the inoculation point of the fruit, the excised samples were flash frozen in liquid nitrogen and stored at −80°C.

Mycelia of P414, R36/14, and 17-21 were grown in clarified V8-juice broth and harvested as described above. Total RNA was extracted from the fruit as described above for strawberry roots and from the flash frozen mycelia of P414, R36/14, and 17-21 as described above. The RNA was assessed by the NanoDrop and Qubit 2 as described above. RNA samples were normalised to 720 ng. Reverse transcription was performed on three biological replicates of each *in planta* time point and two biological replicates for the mycelia timepoints with the QuantiTech Reverse Transcription Kit (Qiagen). *In planta* timepoints, for further analysis, were confirmed through the positive identification of *P. cactorum* β-tubulin product by Reverse Transcription-Polymerase Chain Reaction (RT-PCR).

Reverse Transcription quantitative PCR (RT-qPCR) was then performed in a CFX96^TM^ Real-Time PCR detection system (BioRad) in 10 μL reactions of: 5 μL of 2x qPCRBIO SyGreen Mix Lo-Rox (PCR Biosystems), 2 μL of a 1:5 dilution of the cDNA sample in dH_2_O and 400 nM of each primer (detailed in Supplementary Table 1). Due to the presence of primer dimers, an additional step at the end of the reaction was added to measure the fluorescence at a temperature greater than the melting temperature of the primer dimers (Ball et al., 2003). The reaction was run with the following conditions: 95°C for 2 min, 40 cycles of 95°C for 5 s, 62°C for 20 s and 80°C for 5 s. This was followed by 95°C for 10 s, and a 5 second step ranging from 65 to 95°C by 0.5°C every cycle to generate melt curves. At least two technical replicates for each sample were performed and the melt curves were analysed to ensure the correct product was detected. Relative gene expression was calculated using the efficiency corrected method, which determines the relative gene expression ratio based on the real-time PCR efficiencies and the cycle quantification value (C_q_; Pfaffl, 2001):

$$\begin{array}{l} ratio=\frac{{\left({\text{E}}_{\text{target}}\right)}^{\Delta CP_{\text{target}}\left(\text{control}-\text{sample}\right)}}{{\left({\text{E}}_{\text{ref}}\right)}^{\Delta CP_{\text{ref}}\left(\text{control}-\text{sample}\right)}} \end{array}$$

Expression values were calculated as the mean of the three biological replicates and the standard error of the mean was calculated and plotted. A pooled sample of all cDNA was used as an inter-plate control (IPC) on all plates using primers for β-tubulin (Pcac1_g23639; Supplementary Table 1). Genes of interest were normalised to two endogenous reference genes (Supplementary Table 1), a ribosomal 40S protein (Pcac1_g24902) and a protein of the BAR-domain family, Pc_WS41, Pcac1_g27577 (Yan and Liou, 2006) and were plotted relative to the expression of the gene of interest in mycelia.

## Results

### *Phytophthora cactorum* Isolates Show Specialisation to Strawberry Crowns and Apple

**Figure 1:**
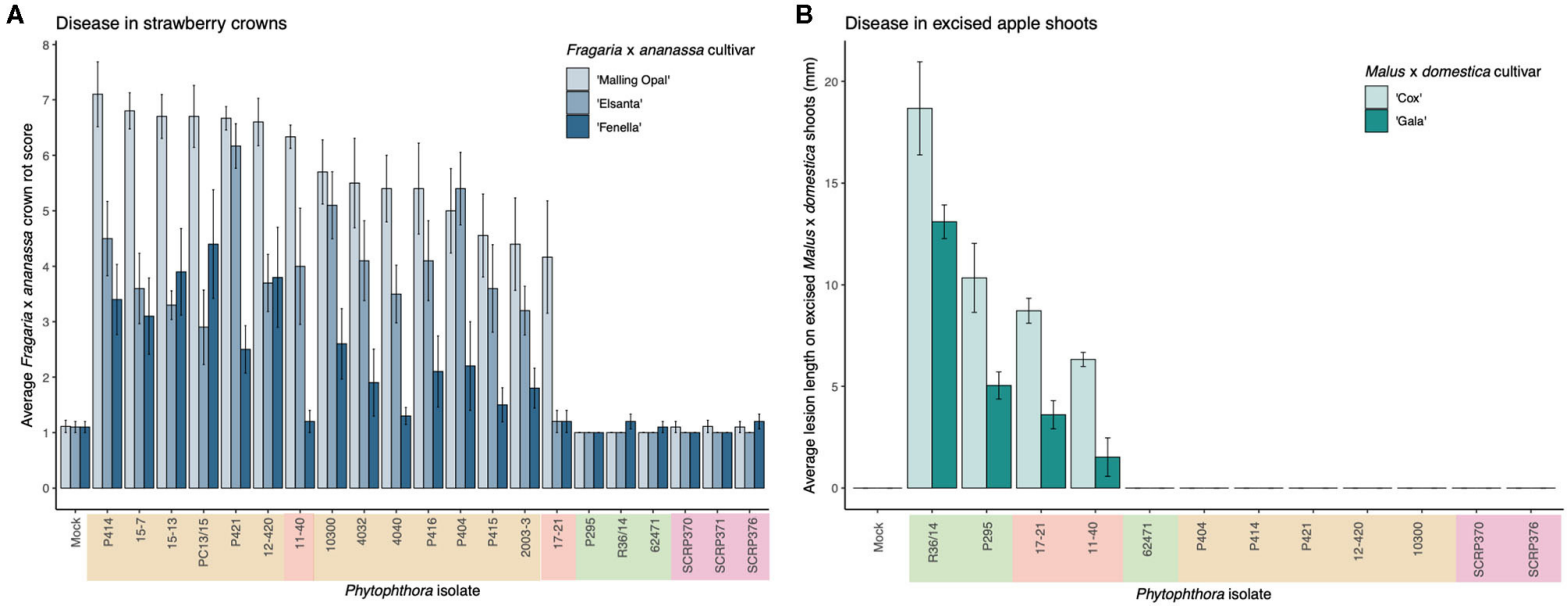
*Phytophthora cactorum* isolates show specialisation to strawberry crown and apple tissue. **(A,B)** Virulence of *P. cactorum* and *P. idaei* isolates on strawberry crowns and excised apple shoots by artificial inoculation of zoospores and mycelium, respectively. The colour behind the isolate names denotes what plant tissue they were cultured from; beige, strawberry crowns, red, strawberry fruit, green, apple bark and purple, raspberry (*P. idaei*). **(A)** Virulence of *Phytophthora* isolates on three *Fragaria* x *ananassa* cultivars, ‘Malling Opal,' ‘Elsanta,' and ‘Fenella.' Data are the mean of ten biological replicates ± se. **(B)** Virulence of *Phytophthora* isolates on two *Malus* × *domestica* cultivars, ‘Cox' and ‘Gala.' Data are the mean of six biological replicates ± se.

A clear difference in pathogenicity on different plant tissues was observed between the isolates from the different *P. cactorum* pathotypes (Figure 1, Supplementary Figure 2). Variation in pathogenicity on strawberry crowns was observed between the *P. cactorum* isolates cultured from strawberry (*p* = 0.02; Figure 1A). All strawberry CR isolates were able to cause disease in strawberry crowns to varying degrees on the three different strawberry cultivars (Figure 1A). Of the two strawberry LR isolates, 11–40 was able to cause disease in both ‘Malling Opal' and ‘Elsanta,' whereas 17-21 was only able to cause disease in the very susceptible ‘Malling Opal' (Figure 1A). None of the apple isolates were able to cause disease in the strawberry crowns (Figure 1A). Variation in pathogenicity on excised apple shoots was observed between the *P. cactorum* isolates cultured from apple and strawberry LR (*p* = 0.03; Figure 1B). Apple isolate R36/14 was found to be the most pathogenic on the shoots (Figure 1B). *P. cactorum* isolate 62471 was shown to be pathogenic on apple seedlings in a previous screen in 2018 (data not shown). Of the two strawberry LR isolates, 17–21 was more pathogenic on the apple shoots than 11–40 (Figure 1B). None of the strawberry CR isolates tested were able to cause disease in apple shoots (Figure 1B). The two strawberry LR isolates appear to have a broader host range than either strawberry CR or apple isolates and are able to cause disease in both strawberry crowns and apple shoots (Figure 1). All representative isolates from the three pathotypes of *P. cactorum* were able to colonise strawberry fruit (Supplementary Figure 2). Isolate 62471 appeared to be the weakest apple isolate as it caused the lowest percentage infection in the strawberry fruit over the three experiments (Supplementary Figure 2), coinciding with its weak pathogenicity on apple tissue. No disease symptoms were recorded in the strawberry tissue or apple tissue when challenged with the *P. idaei* isolates (Figure 1, Supplementary Figure 2). Although, it should be noted that the *P. idaei* isolates were also tested on raspberry fruit but the results were inconclusive.

### Generation of an Improved Contiguous *P. cactorum* Genome

Here we present the best assembly to date of the plant pathogen *P. cactorum*. The SMRT data for the strawberry CR isolate P414 yielded an assembly of 66 Mb in 194 contigs. The other *P. cactorum* isolates, with the exception of P404, yielded *de novo* Illumina assemblies of 59.7–61.6 Mb in 4,452–6,726 contigs (Table 2), with isolate P404 larger and in a greater number of contigs, totalling 75.5 Mb in 20,136 contigs. The *P. idaei* genomes were a similar size to *P. cactorum* assemblies, 60.4–60.6 Mb in 4,720–5,356 contigs. Gene space between all assemblies was comparable, with 224–230 of 234 (95.7–98.3%) BUSCO genes both present and complete in the assemblies (Table 2), which were comparable to previous *Phytophthora* spp. sequencing projects, 91.5–94.4% for *P. cinnamomi* (Longmuir et al., 2018).

**Table 2:** Assembly statistics for 17 *Phytophthora cactorum* and three *Phytophthora idaei* isolates sequenced in this study.

**Features**		***Phytophthora cactorum***																***Phytophthora idaei***		
		***Fragaria*** **×*****ananassa*** **crowns**											***Fragaria*** **×*****ananassa*** **fruit**		***Malus*** **×*****domestica***			***Rubus idaeus***		
		**P414**	**12–420**	**15–13**	**15–7**	**2003–3**	**4032**	**4040**	**P404**	**P415**	**P421**	**PC13/15**	**11–40**	**17–21**	**62471**	**P295**	**R36/14**	**SCRP370**	**SCRP371**	**SCRP376**
Coverage		76.49	25.60	55.63	59.71	69.29	53.14	67.21	69.52	86.64	106.94	77.83	68.90	78.86	72.21	39.29	56.09	40.91	71.89	49.19
No. of contigs		194	5,509	5,421	5,457	5,467	6,726	5,326	20,136	5,695	5,485	5,278	4,571	4,452	6,173	5,088	5,952	5,356	4,720	5,313
Assembly size (Mb)		66.0	59.9	60.7	60.8	60.8	61.6	60.7	75.5	60.8	60.5	60.0	61.1	59.7	59.8	61.3	60.7	60.5	61.7	60.6
Longest contig (bp)		1,749,730	272,591	358,283	300,516	353,051	299,428	238,735	346,862	313,236	251,913	346,832	288,536	347,645	285,881	357,485	345,260	247,223	295,942	230,545
N50 (bp)		645,375	37,498	41,248	43,376	40,273	38,202	39,295	36,598	40,742	34,103	39,779	43,828	49,722	36,408	48,651	36,465	39,576	46,381	39,406
Masked (% bp)		29.2	24.9	26.2	26.0	26.2	26.1	26.1	18.4	26.5	26.0	25.9	24.5	25.1	25.6	25.6	26.2	27.0	26.6	27.3
BUSCO	C	224	228	227	228	228	228	228	227	228	226	228	227	227	226	227	227	230	230	229
	% present	95.7	97.4	97.0	97.4	97.4	97.4	97.4	97.0	97.4	96.6	97.4	97.0	97.0	96.6	97.0	97.0	98.3	98.3	97.9

*Isolate P414 represents results from long read sequencing technologies whereas other assemblies are a result of short read sequencing technology. Information on sequencing coverage, assembly size, percentage of bases repeatmasked are shown. Assembly completeness statistics, assessed using Alveolata-Stramenopiles Benchmarking Universal Single-copy Orthologue (BUSCO) genes, detail the number of complete (C) and total percentage of 234 core Eukaryotic genes present in assemblies*.

### Whole Genome Phylogeny Supports Resolution Between *P. cactorum* Pathotypes

**Figure 2:**
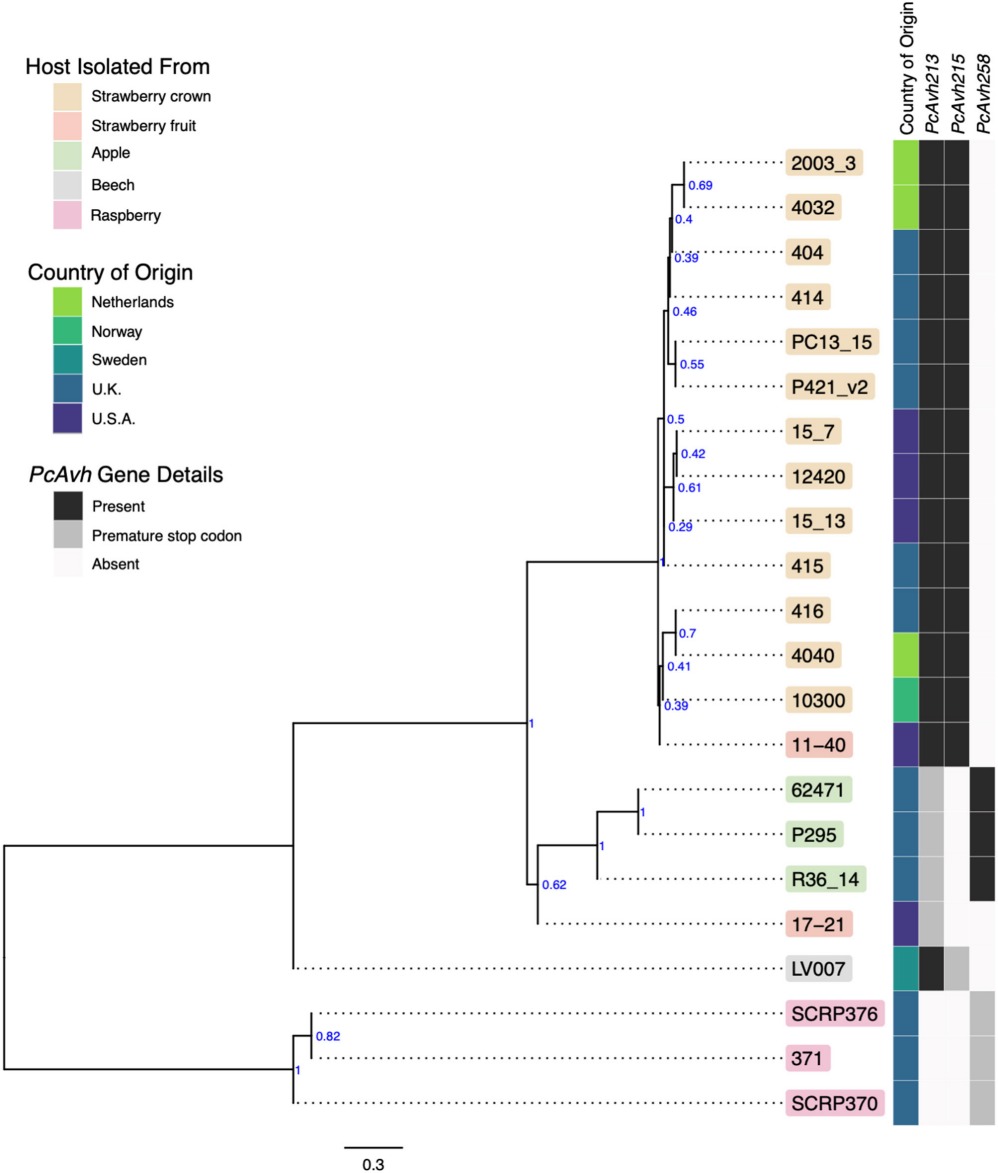
Apple and strawberry crown rot *Phytophthora cactorum* isolates form distinct clades. Maximum parsimony consensus phylogeny of 230 Alveolata-Stramenopiles Benchmarking Universal Single-Copy Orthologue (BUSCO) genes from 22 *Phytophthora* isolates. Branch support shown in blue. Details of the host species isolated from, country of origin and presence/absence of a *Phytophthora sojae Avh32* homologue (*PcAvh215*) and *Phytophthora infestans Avr3a* homologue (*PcAvh258*) and *PcAvh213* are shown in the heatmap.

A consensus phylogeny of 230 conserved single copy genes from the 22 *Phytophthora* isolates showed clear resolution between species, with *P. cactorum* and *P. idaei* isolates resolved into distinct clades (Figure 2). Resolution was also shown within *P. cactorum*, with the 13 strawberry CR isolates, including the previously sequenced CR isolate 10300, present in a distinct clade from the three apple isolates. The two strawberry LR isolates were placed into different clades. Strawberry LR isolate 11–40, which was more virulent on strawberry crowns, was placed in the same clade as the strawberry CR isolates. Whereas, strawberry LR isolate 17–21, which was more virulent on apple, was placed in the same clade as the apple isolates. Interestingly, the publicly available *P. cactorum* isolate from *F. sylvatica* was genetically distinct from all other *P. cactorum* isolates (Figure 2). Within the strawberry clade, no evidence was observed for isolates being associated with geographical distribution, with isolates from the U.K., Netherlands, Norway and U.S.A., observed to be distributed throughout the clade.

### Population Structure Reflects Predominant Asexuality Within Diverging *P. cactorum* Lineages

**Figure 3:**
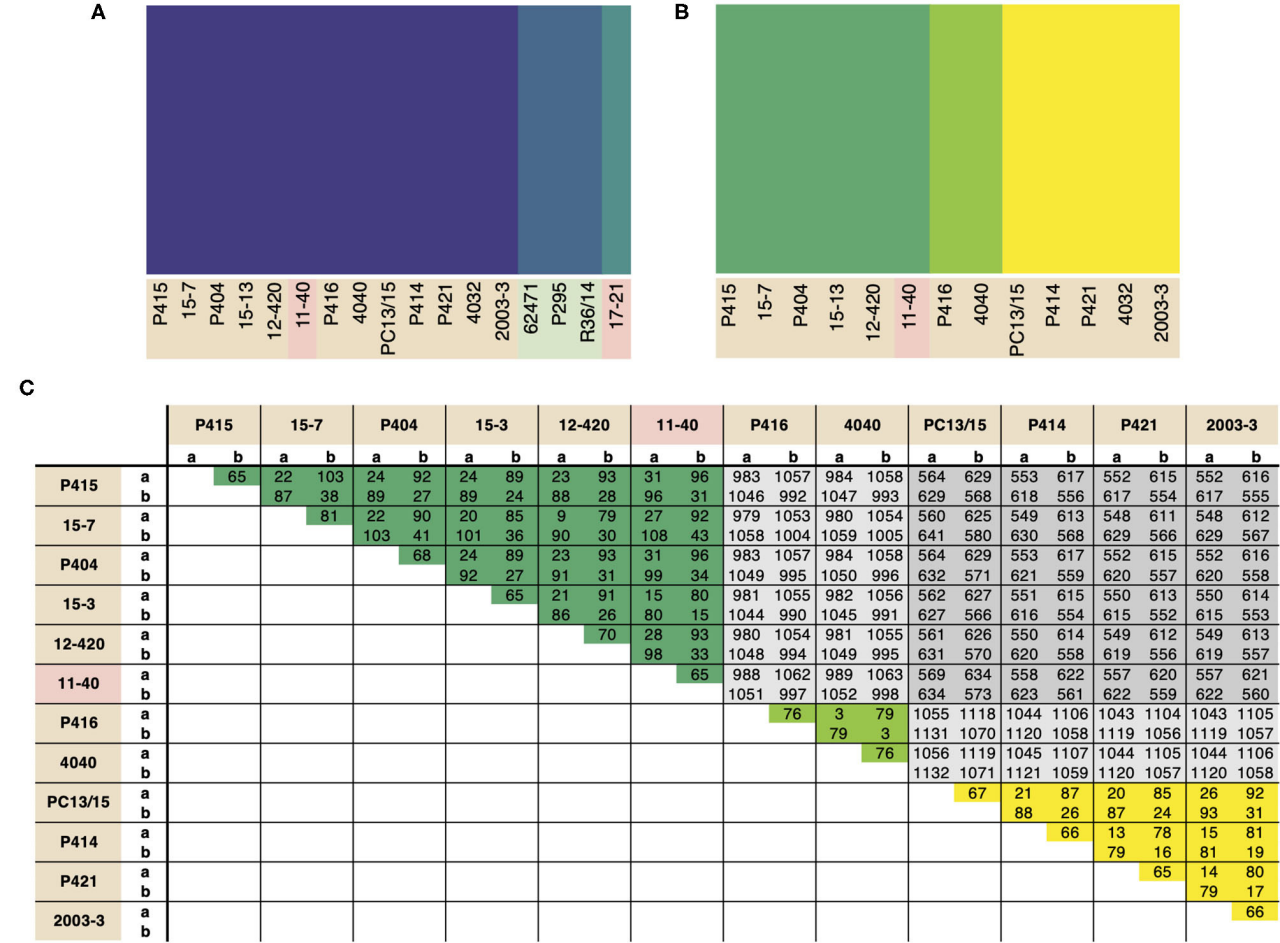
*Phytophthora cactorum* isolates split into three populations with pre-dominant asexuality. Population analysis of high quality, biallelic SNP sites split the *P. cactorum* isolates into three populations, admixture of variants was not observed within *P. cactorum*, or within a single lineage of *P. cactorum* indicating predominant asexuality. DISTRUCT plot of fastSTRUCTURE (Raj et al., 2014) results carried out on **(A)** all sequenced isolates of *P. cactorum* and **(B)** strawberry lineage isolates. Data was best described by three “populations” in both datasets. **(C)** SNP calling was performed in relation to reference isolate P414 for the *P. cactorum* strawberry lineage. The number of differing sites are shown between the two haplotypes from each isolate; a, representing only homozygous variants and b, representing both homozygous and heterozygous variants in relation to the P414 reference genome.

Population structure within *P. cactorum* was investigated using SNP variants predicted in relation to the strawberry CR isolate P414. *P. cactorum* SNP data was found to be best described by three non-recombining populations representing the strawberry lineage, apple lineage and the LR isolate 17–21, respectively (Figure 3A). Proportions of shared variants between these populations was <0.1% in each isolate, indicating a lack of recombination between populations. Subpopulation structure also showed indications of asexuality when restricted to isolates within the strawberry lineage (Figure 3B). Isolates within the three subpopulations of the strawberry lineage showed high genetic identity to one another, with the number of heterozygous sites observed within a diploid individual being close to the genetic distance between two individuals from the same population (Figure 3C). For example, in the subpopulation consisting of 4040 and P416, 44 sites were found to be heterozygous within isolate 4040, whereas the total number of SNPs that showed variability between isolate 4040 and P416 (including heterozygous and homozygous sites) was observed to be 79 (Figure 3C). This was in contrast to the number of SNPs differing between isolates between subpopulations, where isolate 4040 differed by a total of 1,054 SNPs to its next closest isolate, 15–7, from another subpopulation (Figure 3C).

### *P. cactorum* Possesses an Expanded Effector Repertoire in Comparison to *P. idaei*

The predicted proteome of *P. cactorum* CR isolate P414 totalled 29,913 proteins encoded by 29,552 genes. Additional isolates were predicted to carry a similar number of proteins 25,449–29,955 and genes 24,856–29,124, with the exception of P404 (Table 3). Within *P. cactorum*, strawberry and apple isolates had similar numbers of predicted genes and effectors. Despite the larger number of predicted genes, P404 had a comparable number of predicted RxLR, CRN, and apoplastic effectors to the other *P. cactorum* isolates. However, *P. idaei* isolates carried a reduced predicted effector repertoire (Table 3), with fewer secreted carbohydrate active enzymes (CAZYmes), CRNs, Elicitins, necrosis-inducing proteins (NLPs), glucanase inhibitors and kazal protease inhibitors predicted than *P. cactorum* isolates.

**Table 3:** Predicted gene models and effectors.

**Features**		***Phytophthora cactorum***																	***Phytophthora idaei***		
		***Fragaria*** **×*****ananassa*** **crowns**												***Fragaria*** **×*****ananassa*** **fruit**		***Malus*** **×*****domestica***			***Rubus idaeus***		
		**P414**	**12–420**	**15–13**	**15–7**	**2003–3**	**4032**	**4040**	**P404**	**P415**	**P416**	**P421**	**PC13/15**	**11–40**	**17–21**	**62471**	**P295**	**R36/14**	**SCRP370**	**SCRPP371**	**SCRP376**
Genes		29,552	25,444	25,942	25,855	28,950	26,062	28,670	34,978	26,042	28,623	25,566	25,771	28,046	27,869	28,767	25,627	29,124	27,354	24,856	24,924
Proteins		29,913	26,265	26,779	26,646	29,811	26,859	29,557	35,812	26,850	29,425	26,155	26,641	28,851	28,599	29,620	26,368	29,955	27,951	25,449	25,557
Secreted		1,887	1,658	1,686	1,668	1,758	1,707	1,758	2,056	1,719	1,770	1,345	1,697	1,723	1,790	1,771	1,666	1,799	1,610	1,540	1,522
EffectorP		507	472	470	475	525	502	522	660	499	537	381	484	511	532	520	476	543	481	456	443
CWDE		281	222	234	220	222	222	225	261	224	219	215	227	222	237	233	229	217	200	194	195
RxLR		158	134	134	136	136	139	137	135	151	144	135	143	132	144	146	142	146	146	144	138
CRN		127	85	89	88	74	94	88	84	93	88	74	91	70	73	74	93	97	69	59	62
TFs		1,215	904	913	921	1,011	924	978	1,064	946	1,007	861	932	968	952	1,003	928	1,038	905	884	862
MAMPs	Elicitin	43	43	42	46	44	43	47	43	46	43	43	44	44	45	44	44	43	37	32	38
	TGA	14	13	13	13	13	13	13	13	12	13	11	13	12	13	13	13	12	12	12	13
Cutinase	5	5	5	5	5	5	5	5	5	5	5	5	5	5	5	5	5	2	3	2	2
NLP	22	29	29	28	28	28	26	23	29	27	27	26	24	32	32	17	32	11	19	14	14
Pcf	3	3	3	3	3	3	3	3	3	3	3	3	3	3	3	3	3	3	4	4	4
GI	21	15	16	18	14	16	15	15	17	16	14	15	18	17	15	19	13	8	10	11	11
Protease inhibitors	Kasal	17	17	17	17	16	16	15	16	17	16	15	16	17	16	16	16	16	13	15	14
	Cath.	3	3	3	3	3	3	4	4	3	3	3	3	3	3	3	4	3	3	3	3
	Cyst.	2	1	1	1	2	1	2	2	1	2	1	1	2	1	2	1	2	1	1	1

*Total number of gene models and the predicted proteins that they encode are shown. Numbers of secreted proteins, secreted cell wall degrading enzymes (CWDE), RxLR and crinkler (CRN) family cytoplasmic effectors are shown, along with predicted transcription factors (TFs). Host defence triggering (MAMP) family sterol binding (Elicitn) and transglutanimase (TGA) proteins are shown as well as apoplastic effector families including necrosis inducing proteins (NLP), Phytophthora cactorum factor (PcF), and protease inhibitors (kazal-, cathepsin-, and cystatin-acting)*.

### Orthology Analysis Identifies Gene Expansion and Contraction Associated With Phylogenetic Lineage

**Figure 4:**
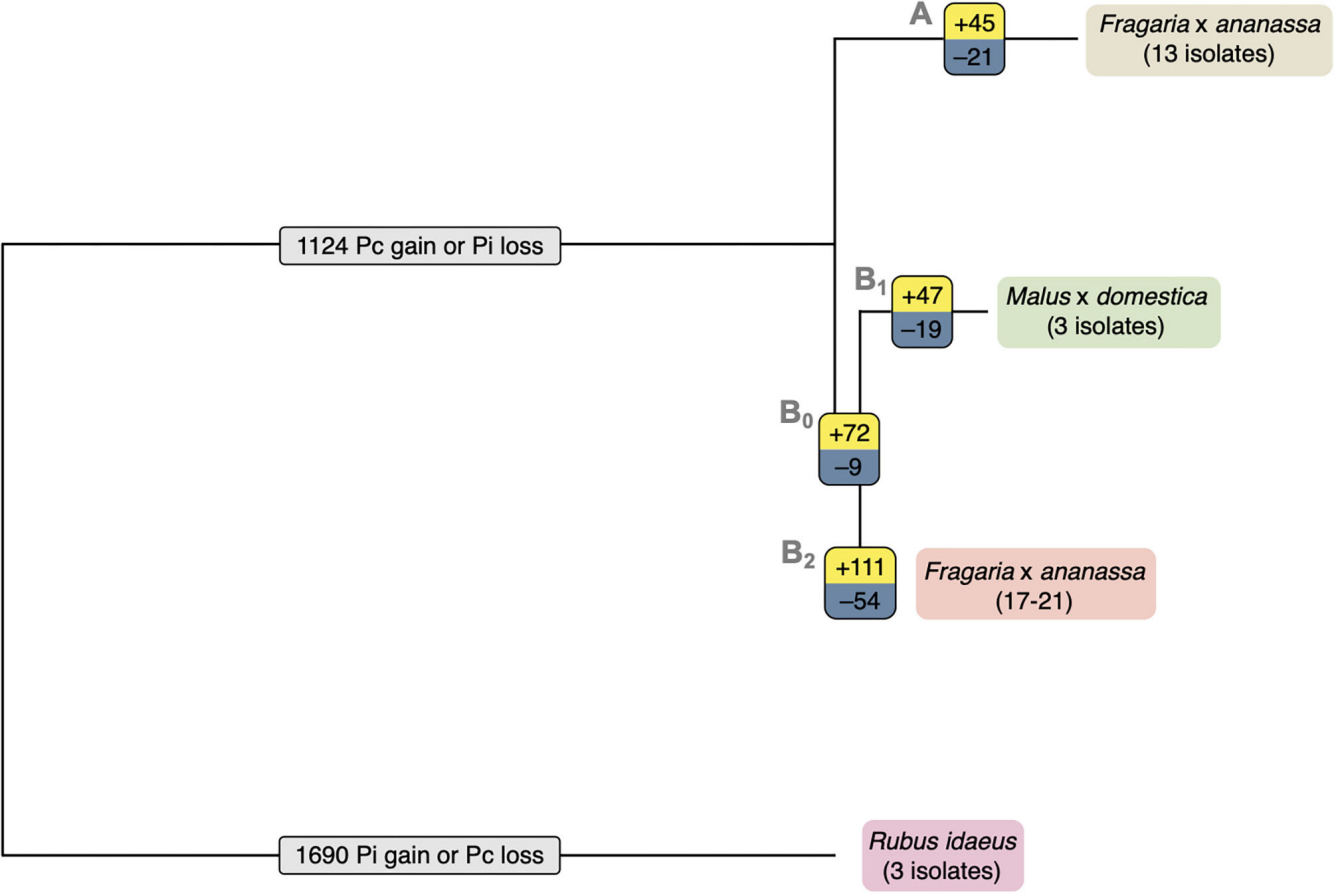
Expansion and contraction of orthogroups indicates possible roles in *P. cactorum* host specialisation. Expansion and contraction of orthogroups plotted onto simple phylogeny. The number of expanded orthogroups of each branch are shown in yellow, the number of contracted orthogroups of each branch are shown in blue and the numbers of orthogroups with unsure direction of expansion or contraction are shown in light grey. The strawberry crown rot lineage (branch A) includes isolates P414, P404, P415, P416, P421, 4032, 4040, 12–420, 2003–3, 10300, 15–7, 15–13, and PC13/15. The apple lineage (branch B_1_) includes isolates R36/14, P295, and 62471.

The total set of 157,038 predicted proteins from the 20 sequenced isolates, as well as the proteome of *P. cactorum* isolate 10300 from Armitage et al. (2018), were clustered into 22,572 orthogroups. Orthogroups showing a consistent pattern of expansion/contraction by phylogenetic clade were identified (Figure 4). This allowed investigation into expansion/contraction events associated with the strawberry CR and apple lineages of *P. cactorum*.

The 45 orthogroups expanded in the strawberry CR lineage (Branch A; Figure 4), represented a total of 65 genes from P414 (Supplementary Table 2). This included a number of potential effector candidates, notably two RxLRs (*Pcac1_g24384* and *Pcac1_g22827*) and an additional secreted protein (*Pcac1_g6287*). BLAST searches confirmed the absence of *Pcac1_g24384* in the apple lineage to be the result of these regions being absent from assemblies rather than genes not being predicted in those genomes. PCR of gDNA from R36/14 and 17-21 did not detect the gene. BLAST searches revealed that isolates in the apple lineage have a region that is homologous to *Pcac1_g22827*, however, there is an indel present in all four isolates. There is an additional G at base 99, in comparison to the strawberry CR lineage, resulting in a frame shift mutation and a premature stop codon at amino acid (aa) 38 and the gene not being predicted in these genomes. The strawberry CR lineage was found to have contracted across 21 orthogroups (Branch A; Figure 4), representing 33 genes in P414 (Supplementary Table 2). This included three RxLRs (*PC123_g16852, PC123_g26877* and *PC123_g27632*) and two additional secreted proteins (*PC123_g10425* and *PC123_g25979*) with effector-like structure that were lost in relation to the wider phylogeny.

The apple isolates and 17–21 lineage harboured greater diversity, with 119 orthogroups expanded (Branches B_0_, B_1_; Figure 4), representing a total of 241 genes (Supplementary Table 2). This included five RxLR (*PC123_g15654, PC123_g17462, PC123_g19522, PC123_g24792*, and *PC123_g25079*) and two CRN (*PC123_g21108* and *PC123_g24736*) candidates, as well as seven secreted proteins (*PC123_g10425, PC123_g10510, PC123_g11377, PC123_g14333, PC123_g24080, PC123_g27245*, and *PC123_g28487*), six of which had an effector-like structure (Supplementary Table 2). BLAST searches confirmed the absence of these genes in the strawberry CR lineage to be a result of these regions being absent from assemblies rather than genes not being predicted in those genomes. A single RxLR (*PC123_g19522*) and CRN (*PC123_g24736*) candidate were identified unique to apple isolates (Branch B_1_; Figure 4). PCR of gDNA of *PC123_g19522* (hereafter *PcAvh258*; all RxLR homologues summarised in Supplementary Table 3) confirmed the absence of this gene in the strawberry isolates P414 and 17–21. *PcAvh258* was found to have 58% pairwise aa homology (downstream of the signal peptide, aa 24–139) to *P. infestans Avr3a* (GenBank AEH27535.1; aa 22-147) (Armstrong et al., 2005). Further investigation of *PC123_g24792* noted a difference between the apple isolate homologues and that in 17–21. The homologue in the three apple isolates was truncated (69 aa; hereafter *PcAch246t*) compared to *PC128_g25726* in 17-21 (195 aa; hereafter *PcAvh246*). A non-synonymous SNP introduced at G210A resulted in a stop codon.

Contraction of 28 orthogroups was observed in the apple lineage (Branches B_0_, B_1_; Figure 4), representing 45 genes. This included only one RxLR candidate (*Pcac1_g13631*) and three additional secreted proteins (*Pcac1_g3068, Pcac1_g3069* and *Pcac1_g25117*), indicating a substantial increase in the effector complement of this lineage.

Overall, these results show that the apple lineage within *P. cactorum*, harbours greater diversity in effector complement than the strawberry CR lineage. RxLRs and CRNs were represented in the expanded and contracted gene families, as well as other unannotated proteins with an effector-like structure. However, other commonly observed *Phytophthora* pathogenicity factors such as secreted CAZYmes, elicitins and protease inhibitors were notably absent from these groups.

### Polarising of SNP and Indel Variants Identifies Putative Host Specialisation Events in Effectors

Further variants were determined through identification of SNP, indel and small structural variants (insertions and duplications) from all sequenced isolates in comparison to strawberry CR isolate P414 (Table 4). Polarising of non-synonymous SNPs and indels to the outgroup *P. idaei* allowed identification of those variants that differed at the species level (private to *P. cactorum*), at the pathotype level (private to apple or strawberry CR isolates), or at the population level. Those variants at the pathotype level were investigated to identify potential signatures of host adaptation. In total, variants were observed in 21 RxLR and 12 CRN genes at the pathotype level (Supplementary Table 4). Of the RxLRs, eight genes contained non-synonymous variants unique to strawberry isolates and 13 unique to apple isolates. Of the CRNs, six genes contained non-synonymous variants unique to strawberry isolates and eight unique to apple isolates (with two genes containing unique variants in both), which in addition to the gene family expansion/contractions described above potentially represent host adaptation events or determinants of host boundaries between pathotypes, or simply functionally neutral mutations fixed due to drift.

**Table 4:** Variant calls vs. the reference P414 genome.

	***P. cactorum***					***P. idaei***
	**P414**	**CR isolates**	**Apple isolates**	**LR 11–40**	**LR 17–21**	**Raspberry isolates**
Total SNPs	67	1,536	26,333	631	20,482	306,259
Gene SNPs	46	844	13,787	357	10,970	165,226
CDS SNPs	30	748	12,318	304	9,701	147,237
**Non-syn/syn SNPs**						
Total	17/13	467/281	6,400/5,918	182/122	4,481/5,220	75,340/71,897
Busco CEGs	0/0	4/11	77/119	2/7	68/90	978/1,453
RxLR	0/0	0/0	15/9	0/0	14/3	299/137
CRN	0/0	0/0	6/2	0/0	7/3	74/35
Total InDels	712	1,760	11,383	1,057	9,200	63,362
Gene InDels	162	394	2,249	241	1,791	14,005
CDS InDels	131	316	1,659	191	1,299	9,282
Busco CEG InDels	0	1	6	0	6	64
RxLR InDels	0	2	12	1	14	81
CRN InDels	2	5	12	2	6	32
Total SVs	0	12	16	0	16	92
Gene SVs	0	8	9	0	6	33
CDS SVs	0	8	9	0	6	29
Busco CEG SVs	0	0	0	0	0	0
RxLR SVs	0	0	0	0	0	0
CRN SVs	0	0	0	0	0	0

*Numbers of SNPs, insertion/deletion events (InDels) and structural variants predicted in relation to strawberry CR P414. Variants leading to changes within gene models and leading to coding changes in CDS are shown. SNP calls within CDS include the numbers of synonymous/non-synonymous variants*.

### Putative Effectors Are Highly Represented in DEGs During Infection of Strawberry

**Figure 5:**
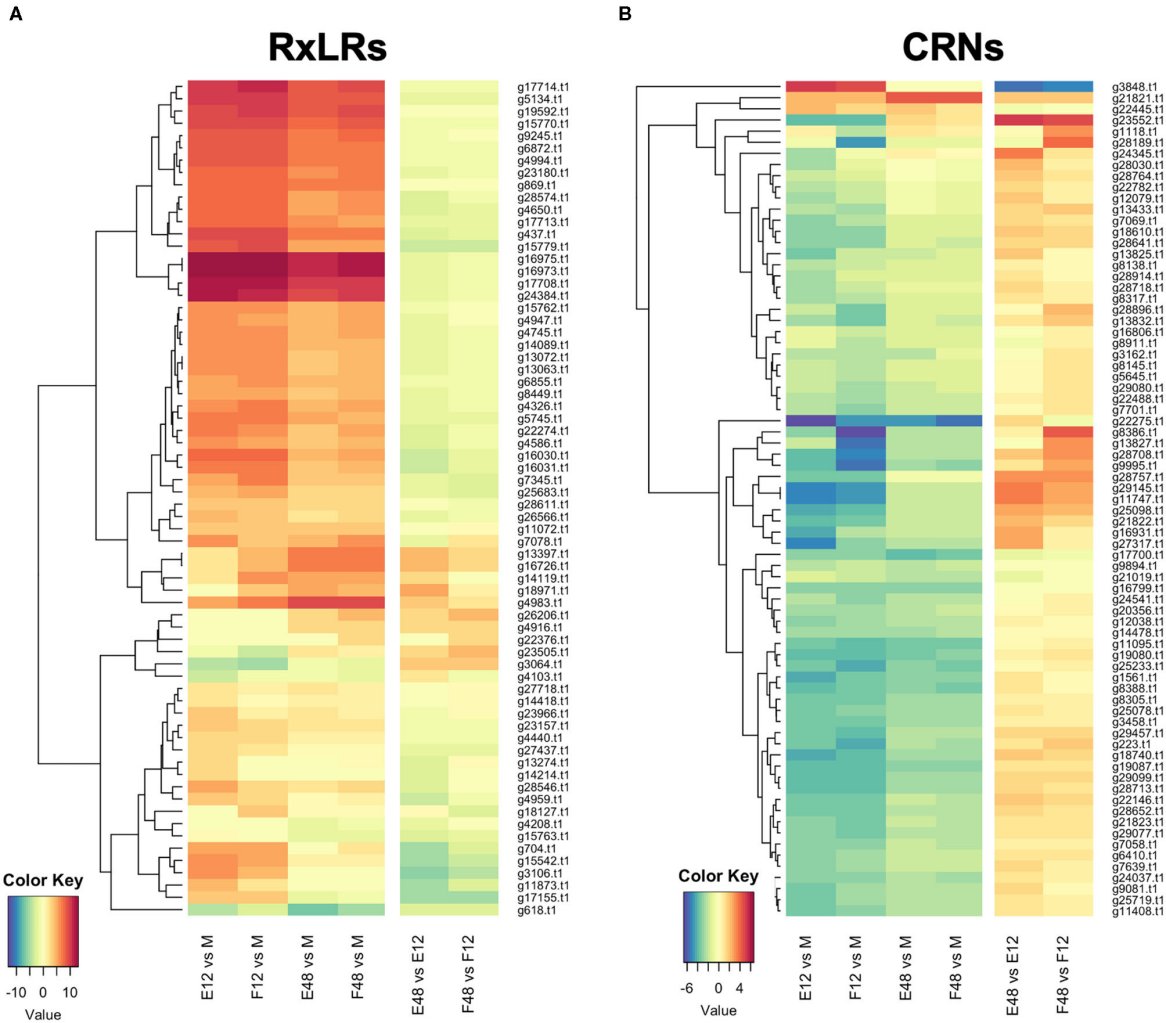
Expression pattern of effectors observed upon infection of both susceptible (‘Emily') and resistant (‘Fenella') strawberry roots. RxLR and crinkler (CRN) genes clustered by expression profile. All gene IDs related to the P414 genome (*Pcac1_*). E, ‘Emily;' F, ‘Fenella;' M, mycelia; 12, 12 hours post-inoculation (hpi); 48, 48 hpi. **(A)** RxLR genes shown in dark red were in the top 25 expressed genes at 12 hpi ranked by log fold change (LFC), **(B)** the time points investigated don't appear to have captured differential expression of CRNs.

RNAseq analysis of *P. cactorum* (P414) infecting strawberry at 12 and 48 hpi showed predicted gene models of putative effectors were upregulated during strawberry infection. Differential gene expression was calculated between mycelium, 12 and 48 hpi in both ‘Emily' and ‘Fenella' cultivars and between 12 and 48 hpi timepoints for both cultivars. This allowed identification of early and late expressed transcripts and of the remaining transcripts, identification of those up- and down-regulated *in planta*. In total 9,178 transcripts with LFC >2 were identified. This equated to 34% of the total transcripts predicted in the genome (Table 5). Putative apoplastic and cytoplasmic effectors were overrepresented within the DEGs with 43–76% of candidates differentially expressed in the experiment. Of these, many secreted CAZYme and RxLR candidates showed temporal expression, showing differential expression only the early or late timepoint (160 and 63 transcripts, respectively), with CAZYmes showing a greater number of late expressed candidates and RxLRs showing greater numbers of early expressed candidates (Table 5, Figure 5A). Furthermore, transglutaminase candidates and Kazal-type protease inhibitors were expressed during early infection, whereas NLP candidates showed a bias towards later infection. Two homologues of *P. infestans* INF1 showed consistent expression across the timepoints (Supplementary Table 4; *Pcac1_g22873* and *Pcac1_g22879*). Interestingly, effectors from each category were identified as down-regulated *in planta*, particularly cytoplasmic CRN effectors, of which 69 were down-regulated at both 12 and 48 hpi (Table 5, Figure 5B). In total, of the 158 putative RxLR effectors identified in P414, just over half, 86 were not expressed or showed low-expression *in planta* (with FPKM values <20) in the RNAseq experiment.

**Table 5:** Expression profile of effector candidates.

	**Early induced**	**Late induced**	**Upregulated *in planta***	**Downregulated *in planta***	**Other DE^a^**	**Total in genome**	**% DEGs of total^b^**
Total transcripts	437	809	2,613	5,319	1,117	29,913	34.4
Secreted	80	141	442	351	91	1,887	58.6
MAMP: elicitin	4	6	23	13	5	67	76.1
MAMP: transglutaminase	4	0	4	3	1	18	66.7
Apoplastic: secreted CAZYmes	19	36	105	40	14	281	76.2
Apoplastic: cutinase	1	0	3	0	0	6	66.7
Apoplastic: glucanase inhibitor	1	1	10	4	0	30	53.3
Apoplastic: NLP	2	6	10	3	0	35	60.0
Apoplastic: phytotoxin	0	0	1	1	0	3	66.7
Apoplastic: protease inhibitor (cathepsin)	0	0	0	2	1	4	75.0
Apoplastic: protease inhibitor (cystatin-like)	0	0	2	0	1	5	60.0
Apoplastic: protease inhibitor (Kazal-type)	5	1	6	4	0	23	69.6
Cytoplasmic: RxLR	9	6	48	4	1	158	43.0
Cytoplasmic: CRN	1	1	2	69	2	127	59.1

*Numbers of differentially expressed (DE) transcripts, summarised by effector family, showing DE at 24 hpi (early induced) or 48 hpi (late induced) in both Fragaria x ananassa ‘Emily' and ‘Fenella' cultivars, or upregulated at both timepoints or downregulated at both timepoints in planta*.

a *DE, Differential expression*.

b *DEGs, Differentially expressed genes*.

### The Most Upregulated Genes *in planta* Include a Broad Range of Effector Candidates

Genes involved in initial establishment of infection were further investigated through ranking transcripts by LFC at 12 hpi in comparison to mycelium in the susceptible host ‘Emily' (all data can be found in Supplementary Table 4). In the top 100 ranked genes (LFC values of 16.8–8), secreted proteins were highly represented with 56 present, including 15 RxLR candidates (along with two additional candidates, that carried RxLR motifs but lacked EER motifs), 13 secreted CAZymes (AA7, CBM63, CE8, GH12, GH28, and PL3 families), four NLP candidates, three kazal-type protease inhibitors (one of which was homologous to *P. infestans* EPI10), two glucanase inhibitors and *P. cactorum* factor (PcF) homologue phytotoxin.

**Figure 6:**
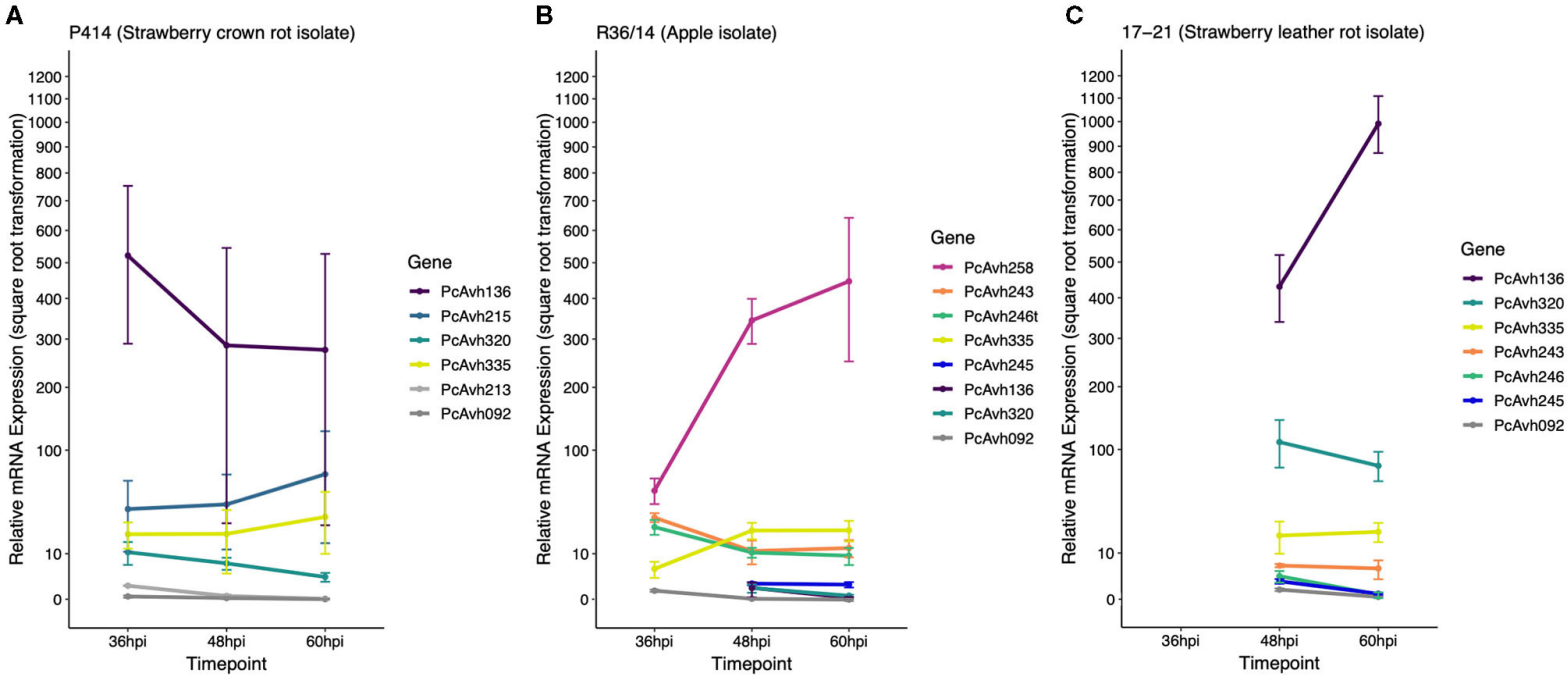
P414, R36/14, and 17–21 RxLR genes show differential expression upon infection of strawberry fruit. **(A–C)** Relative expression of a selection of candidate RxLR genes in three representative *Phytophthora cactorum* isolates. The same gene from different genomes are indicated by the same colour. Genes of interest were normalised to two endogenous reference genes, a ribosomal 40S protein and a protein of the BAR-domain family, Pc_WS41 (Yan and Liou, 2006) and were plotted relative to the expression of the gene of interest in mycelia. Data are the mean of three biological replicates ± se. **(A)** *P. cactorum* isolate P414, strawberry crown rot pathotype representative, **(B)** *P. cactorum* isolate R36/14, apple pathotype representative, **(C)** *P. cactorum* isolate 17–21, strawberry leather rot pathotype representative.

Focusing on the top 25 ranked genes (summarised in Table 6), six RxLR candidates were upregulated upon infection of strawberry (*Pcac1_g16973, Pcac1_g16975, Pcac1_g17708, Pcac1_g24384, Pcac1_g5134*, and *Pcac1_g17714*) and an additional one (*Pcac1_g998*), which carried an RxLR motif without an EER motif. *Pcac1_g24384* (hereafter *PcAvh215*) was of particular interest as it was identified as unique to *P. cactorum* strawberry CR isolates and highly expressed *in planta*. Subsequent RT-qPCR analysis in strawberry fruit supported the findings of the RNAseq timepoints and showed expression of *PcAvh215* (Figure 6A). Notably, this was the only example of a gene private to strawberry CR isolates in the top 100 ranked transcripts. Investigation into this gene showed it to be a homologue of *P. parasitica* XM_008912329 and *P. sojae Avh32* (JN253712; paralogue to *Avh6;* Wang et al., 2011) with 86 and 82% pairwise aa identity, respectively, downstream of the signal peptide (26-147 aa). *Pcac1_g16973/5*, hereafter *PcAvh136*, was a gene duplication event. *Pcac1_g5134* was the only one out of eight RxLRs with a unique polymorphism (non-synonymous SNP, G53A) between strawberry CR isolates and the three apple and 17-21 isolates, that was expressed.

**Table 6:** The most upregulated genes in ‘Emily' at 12 h post infection include a broad range of effector candidates.

**LFC**	**Expression pattern**	**Transcript ID**	**Contig**	**Orthogroup**	**Presence by group**	**Presence variation at level:**	**Non-Syn SNP/indel variation at level**	**Secretion evidence**	**Function annotation**
16.8	Early	g20321.t1	Contig_57	Orthogroup89	CR(14)LR(1)Md(3)Ri(3)				
15.6	Early	g2828.t1	Contig_4	Orthogroup15102	CR(10)LR(1)Md(2)Ri(0)	^*^		Phobius	Coil domain
15.5	Early	g9289.t1	Contig_18	Orthogroup16991	CR(7)LR(0)Md(0)Ri(0)	^*^			
15.3	Early	g12985.t1	Contig_29			^*^			Coil domain
14	Early	g7826.t1	Contig_14	Orthogroup15950	CR(9)LR(0)Md(1)Ri(0)	^*^	Species		
13.6		g12191.t1	Contig_26	Orthogroup359	CR(14)LR(1)Md(3)Ri(3)			SignalP; Phobius	EffectorP; Jacalin-like lectin
13.1		g998.t1	Contig_2	Orthogroup774	CR(14)LR(1)Md(3)Ri(3)			SignalP; Phobius; TM domain	RxLR (no EER motif); EffectorP
13.1	Early	g24044.t1	Contig_79	Orthogroup14845	CR(11)LR(1)Md(2)Ri(0)	^*^	Species		
13		g16973.t1	Contig_43	Orthogroup12686	CR(14)LR(1)Md(3)Ri(0)	*P. cactorum* private		SignalP; Phobius	RxLR; EffectorP
13		g16975.t1	Contig_43	Orthogroup12686	CR(14)LR(1)Md(3)Ri(0)	*P. cactorum* private		SignalP; Phobius	RxLR; EffectorP
12.2		g12202.t1	Contig_26	Orthogroup359	CR(14)LR(1)Md(3)Ri(3)		Species	SignalP; Phobius	EffectorP; Jacalin-like lectin
11.9		g17708.t1	Contig_46	Orthogroup9680	CR(14)LR(1)Md(3)Ri(3)		Species	SignalP; Phobius	RxLR
11.5		g26017.t1	Contig_95	Orthogroup11598	CR(14)LR(1)Md(3)Ri(3)		Species	SignalP; Phobius	
11.4		g24384.t1	Contig_81	Orthogroup14979	CR(14)LR(0)Md(0)Ri(0)	Strawb. CR private		SignalP; Phobius	*PsAvh32* homologue; RxLR; EffectorP
11.2		g20304.t1	Contig_57	Orthogroup947	CR(14)LR(1)Md(3)Ri(3)				Antibiotic biosynthesis monooxygenase
11.2		g20307.t1	Contig_57	Orthogroup947	CR(14)LR(1)Md(3)Ri(3)				Antibiotic biosynthesis monooxygenase
11.1	Early	g7647.t1	Contig_14	Orthogroup3422	CR(14)LR(1)Md(3)Ri(3)		Species		Coil domain
11.1		g9964.t1	Contig_19	Orthogroup13882	CR(14)LR(1)Md(2)Ri(0)	^*^	Species	SignalP; Phobius	Coil domain
10.8		g613.t1	Contig_1	Orthogroup12809	CR(14)LR(1)Md(3)Ri(0)	*P. cactorum* private	Species	SignalP; Phobius	
10.7	Early	g4491.t1	Contig_7	Orthogroup201	CR(14)LR(1)Md(3)Ri(3)				Coil domain
10.7		g13303.t1	Contig_30	Orthogroup8651	CR(14)LR(1)Md(3)Ri(3)		Pathotype, species	SignalP; Phobius; TM domain	EffectorP
10.5		g5134.t1	Contig_8	Orthogroup6597	CR(14)LR(1)Md(3)Ri(3)		Pathotype, species	SignalP; Phobius; TM domain	RxLR
10.3		g10084.t1	Contig_20	Orthogroup1931	CR(14)LR(1)Md(3)Ri(3)			SignalP; Phobius	EffectorP
10.3	Early	g15153.t1	Contig_36	Orthogroup9085	CR(14)LR(1)Md(3)Ri(3)		spec ies	SignalP; Phobius	Protease inhibitor (Kazal-type)
10.3		g15397.t1	Contig_37	Orthogroup1297	CR(14)LR(1)Md(3)Ri(3)		pat hotype, population	SignalP; Phobius	Unamed family (PTHR34737:SF2)
10.3		g17714.t1	Contig_46	Orthogroup9681	CR(14)LR(1)Md(3)Ri(3)			SignalP; Phobius	RxLR

*Transcripts were ranked by log fold change (LFC) in descending order. Gene families expanded, private to Phytophthora cactorum, or private to the strawberry crown rot (CR) lineage were identified. Functional annotation of proteins provide evidence for effector candidates*.

* *denotes orthogroups that could not be tested*.

Looking further afield to the remaining nine RxLRs in the top 100 genes, there was another gene duplication event, *Pcac1_g16030/1*, hereafter *PcAvh320*. *PcAvh320* was identified to be a homologue to *PiAvrblb2* (Oh et al., 2009). This homologue was identified as present in all *P. cactorum* isolates. Subsequent RT-qPCR of P414 supported the RNAseq data and showed expression of the gene upon infection of strawberry fruit (Figure 6A). *Pcac1_g15770* (hereafter known as *PcAvh266/7/8*) was also identified to be a homologue to another known Avr gene, *PiAvrSmira1* (Rietman et al., 2012).

### Unique Strawberry CR Putative Secreted Proteins Are Not All Expressed During Strawberry Infection

Of the three putative secreted proteins expanded in strawberry CR isolates, only RxLR candidate *PcAvh215* was found to be differentially expressed by P414 in the time points investigated in strawberry *in vitro* plants (with peak FPKM values of 3,364 and 4,375 in ‘Emily' and ‘Fenella,' respectively), and strawberry fruit (Figure 6A). The other RxLR candidate, *Pcac1_g22827* (hereafter *PcAvh213*), showed low levels of constitutive expression in mycelium with a peak FPKM value of 6 but was not expressed at any time point *in planta* in the RNA-Seq experiment nor subsequent *in planta* RT-qPCR experiment (Figure 6A). The uncharacterised secreted protein (*Pcac1_g6287*) had a peak FPKM value of 20 *in planta* but was not investigated in the subsequent RT-qPCR.

### Candidate RxLR Genes and Homologues to Known Avirulence RxLR Genes Show Differential Expression Between Isolates

RT-qPCR analysis of a selection of apple lineage specific (Branch B_0_; Figure 4) RxLR candidates show they are expressed by the apple pathotype representative R36/14 upon infection of strawberry fruit (Figure 6B), but not the strawberry LR representative isolate 17–21 (Figure 6C). The truncated *PcAvh246t* in R36/14 was expressed (Figure 6B) but the full-length gene, *PcAvh246*, in 17–21 was not expressed very highly (Figure 6C). *PC123_g25079* (hereafter *PcAvh243*) had a similar pattern of expression in R36/14 to *PcAvh246t* but was more highly expressed than *PcAvh246* in 17–21. *PC123_g17462* (hereafter *PcAvh245*) was not expressed very highly in either R36/14 or 17–21 at the time points investigated. *PC123_g15654* was not investigated by RT-qPCR. *PcAvh258*, the only RxLR candidate unique to apple isolates, was the highest expressed RxLR investigated in isolate R36/14 on strawberry fruit (Figure 6B).

The expression profiles of a selection of known *Avr* genes were investigated during strawberry fruit infection by the representative isolates. A homologue to *PiAvramr1* (Lin et al., 2020; *PcAvh335*) was universally expressed in the three isolates investigated (Figure 6, Supplementary Table 5). In contrast, a homologue to *PiAvramr3* (Lin et al., 2019; *PcAvh092*) was not expressed by any of the isolates at the time points investigated (Figure 6, Supplementary Table 5). A homologue to *PiAvrblb2* (Oh et al., 2009; *PcAvh320*) was shown to have the greatest upregulation in the strawberry-infecting isolates P414 and 17–21, with lower upregulation in R36/14 (Figure 6, Supplementary Table 5). P414 was predicted to have two copies of the *PiAvrblb2* homologue adjacent to each other on contig 39. Other homologues identified but not investigated with RT-qPCR included *PiAvrvnt1* (Pel, 2010; *PcAvh428/9*) which was not expressed by P414 in the RNAseq data and *PiAvrSmira1* (Rietman et al., 2012; Pc366/7/8) which was found to be expressed *in planta* from the RNAseq data. *PcAvh258*, which was only found in apple isolates, was found to be a homologue to *PiAvr3a*. Other homologues not investigated by RT-qPCR are detailed in Supplementary Table 5.

The RNAseq data from strawberry showed candidate *PcAvh136* was highly expressed by P414 *in planta* and was investigated further in the three representative isolates, where it was found to be the highest expressed RxLR effector investigated in both P414 and 17-21 in strawberry fruit by RT-qPCR. The gene although present in the R36/14 genome was not expressed by the apple isolate, and therefore appears to only be expressed by the two strawberry infecting isolates. In addition, candidate *PcAvh320* appears to be expressed higher by P414 and 17-21, compared to R36/14.

## Discussion

Understanding the pathogenicity of plant pathogens is necessary for designing and implementing durable resistance strategies. Pathogen-host interactions are notoriously dynamic and *Phytophthora* spp. exhibit rapid adaptability to host immunity (Wang and Jiao, 2019; Chepsergon et al., 2020). *P. cactorum* is a continuing threat to strawberry and apple production, as well as more generally to forest trees and woody perennials. Our study revealed that isolates of *P. cactorum* have clear differences in pathogenicity and are generally specialised to different hosts. Sequencing of the contemporary isolates of strawberry CR revealed that there is clear separation between the *P. cactorum* isolates infecting strawberry crowns and apple. We determined that although isolates of *P. cactorum* causing strawberry CR are genetically similar globally, variation in virulence between the isolates was observed, especially on host cultivars with a higher level of resistance. Furthermore, our results highlight several RxLRs that warrant further investigation as host specificity determinants for strawberry and apple.

*P. cactorum* is often described as a generalist pathogen with a wide host range (Grenville-Briggs et al., 2017; Yang et al., 2018). However, our results support previous reports (Seemüller and Schmidle, 1979) which showed that *P. cactorum* isolates originating from strawberry crowns are specialised and less pathogenic on apple bark tissue than isolates originating from strawberry fruit or apple. Conversely Seemüller and Schmidle (1979) showed that apple and strawberry fruit isolates were less pathogenic on strawberry crowns. Our data showing the segregation of the strawberry CR and apple isolates into different clades demonstrates clear patterns of separation, which are backed up by analysis of population structure, potentially indicating a species complex and not a single *P. cactorum* species. Of note, the two strawberry LR isolates from our study appear to have a broader host range than either strawberry CR isolates or apple isolates and are able to cause disease in both strawberry crowns and apple excised shoots but to a more limited extent than most apple isolates. Interestingly, these isolates fall both within the clonal strawberry CR lineages and the more diverse “apple” clade. This may be what has led to confusion in the past, as it is clear that some isolates have a slightly broader host range, but potentially at the expense of enhanced virulence on a single host. However, from our work it is hard to draw conclusions from the strawberry LR isolates as only two isolates were investigated in this study. Further investigation of the isolates sequenced in this study through further RNAseq analysis of infection and the collection of additional isolates may provide a greater understanding of the components required to be a successful pathogen on both strawberry and apple. Screening all isolates on additional potential hosts was not part of the scope of this work but would be an appropriate next step to determine the host range of the isolates.

Clonal pathogen populations are a threat to global food security, as the advantageous stabilising of favourable multi-locus associations results in the rapid spread of specialised lineages within susceptible host populations. These populations can readily adapt to new introduced resistance genes, despite the proportionately moderate levels of standing genetic diversity (Stukenbrock and Bataillon, 2012), presumably due to their high census population sizes (Barton, 2010). In asexual lineages of plant pathogens such as *P. cactorum*, very little is known about the degree to which genetic variation within these lineages influences the virulence profile of populations. We observed a lack of SNP diversity between the strawberry CR isolates, despite isolates being collected from multiple countries, across multiple years, indicating clonality and the possibility of a recent bottleneck. Despite the lack of genetic diversity, substantial variation in pathogenicity on ‘Elsanta' and ‘Fenella' strawberry crowns was observed between the strawberry CR isolates. Further investigation is needed to understand whether this variation is either the result of what little genetic variation there is, or whether there is also stably inherited epigenetic silencing of effectors, which has recently been reported in *P. sojae* (Wang et al., 2019) or DNA methylation. N6-methylation (6 mA) was profiled in both *P. infestans* and *P. sojae*, however, its exact role in the regulation of gene expression remains to be fully examined (Chen et al., 2018). In the distantly related *P. fragariae*, another pathogen of strawberry, our previous work highlighted that variation in virulence was not related to DNA sequence variation but rather differences in the expression of putative effectors was associated with race structure (Adams et al., 2020). It was proposed that silencing of *Avr* RxLR effectors enables isolates to evade recognition in plants possessing the corresponding *R* gene. The exact mechanism of silencing was not determined during the study, but we postulated that variation was attributed to either control in *trans* or stable forms of epigenetic modifications were regulating gene expression (Adams et al., 2020). Little is known about how filamentous pathogens adapt to host plants by factors other than DNA sequence polymorphism. This study revealed that over half (86) of our effector candidates were not expressed or showed low-expression *in planta*. This indicates that there is pervasive silencing of effectors, highlighting the crucial role of RNAseq data when attempting to determine effector candidates for onward study.

Non-host resistance (NHR), described roughly as the ability of a plant species to ward off the colonisation of all genotypes of a pathogen species, is poorly understood but often considered the most durable form of resistance, due to the large number of independent resistance mechanisms that are likely acting. Studies in a wide range of pathogens provide evidence for the role of secreted effectors in determining host range (Lee et al., 2017). A classic example is the deletion of a single effector gene, *hopQ1-1* in *Pseudomonas syringae* pv. *tomato*, enabling the pathogen to extend its host range within the Solenaceae to the non-host plant species, *Nicotiana benthamiana* (Wei et al., 2007). However, non-host resistance ranges from single effectors controlling host range to potentially many tens of effectors. A study of 54 *P. infestans* RxLR effectors in the non-host pepper uncovered that up to 36 could be recognised and led to hypersensitive response in some accessions, suggesting that the recognition of multiple *P. infestans* effectors leads to NHR (Lee et al., 2014). This large number of recognised effectors may be due to the evolutionary differences between wild potato and *Capsicum* species – reported to have split around 20 Ma (Särkinen et al., 2013), which although geographically overlapping have distinct ecological niches (Rumold and Aldenderfer, 2016; Barboza et al., 2019). However, it is possible that *Phytophthora* isolates may carry within their genome many more effectors than they actually express (as we highlight above) and so what matters is which effectors are expressed during infection. What also matters is not just the antigenic potential of the effectors that are expressed, but the function of the effector network in the infection process. It is well-known that some effectors are able to mask the avirulence potential of other effectors and therefore evidence for activation of the hypersensitive response alone is also insufficient to assess the functional consequence of a lineage possessing any single effector (Derevnina et al., 2021).

From our work it is not possible to tell how many effectors are causing the observed differences in host range between the clonal CR lineage and isolates infective on apple. The reciprocally incompatible interactions that we observe could be due to effectors either being recognised or the inability of the lineage to efficiently manipulate the host. However, our work did identify candidate RxLR's which may be good candidates for host specificity determinants on strawberry and apple. Two candidate host-range determinants on strawberry crowns and apple that warrant further investigation are PcAvh215 and PcAvh258, respectively. The strawberry LR isolate, 17–21 does not possess PcAvh215 or PcAvh258 but is pathogenic on both strawberry and apple, to a limited extent compared to isolates recovered from the specific host tissue. This discounts these effectors from being solely responsible for pathogenicity on either host, but they possibly enable the isolates possessing them to have greater virulence on the respective host. Candidate effector *PcAvh136* was found to be expressed by both the strawberry CR isolate P414 and the strawberry LR isolate 17–21, but not the apple isolate R36/14 upon infection of strawberry fruit, indicating it may possibly be a determinant for pathogenicity on strawberry. It may also be that *PcAvh215* enables isolates possessing it to be more pathogenic on strawberry crowns (all strawberry CR isolates and strawberry LR isolate 11–40), but further RNAseq and transformation of it into non-PcAvh215 containing isolates such as 17–21 would be required to investigate this hypothesis further. Of the effectors that were found to be polymorphic between apple and strawberry only one showed evidence for expression in strawberry.

Understanding effector profiles has aided the characterisation of resistance genes (Armstrong et al., 2005; Poppel et al., 2008; Champouret et al., 2009; Oh et al., 2009; Gilroy et al., 2011; Rietman et al., 2012; Sugimoto et al., 2012), and thereby provided an insight into resistance durability in the field. Homologues to major gene targets (homologues to known *Avr* genes) that are expressed by *P. cactorum* include, *PiAvr1* (Champouret et al., 2009) *PiAvrblb1* (Champouret et al., 2009), *PiAvrblb2* (Oh et al., 2009), *PiAvrSmira1* (Rietman et al., 2012) and *PiAvramr1* (Lin et al., 2020). It would be interesting to investigate if homologues to the resistance genes *Rpiblb1, Rpiblb2*, and *RpiSmira1* are involved in resistance to *P. cactorum* in strawberry and apple. In addition, there are multiple major gene resistance targets in *P. cactorum* that are present but were not expressed in the *in planta* RNAseq time course of the strawberry CR isolate P414, for example, *PiAvrvnt1* (Pel, 2010), *PiAvr4* (Poppel et al., 2008), *PiAvr8/PiAvrSmira2* (Rietman et al., 2012), and *PiAvramr3* (Lin et al., 2019), it is unknown if these play a role in virulence in *P. cactorum*.

Copy number variation (CNV) is also another form of genetic adaptation that has been identified in *Phytophthora* genomes (Qutob et al., 2009). For example, *P. sojae, P. parasitica*, and *P. infestans* genomes encode at least 2, 8, and 11 *Avrblb2* homologues, respectively (Naveed et al., 2019). In the most contiguous *P. cactorum* genome of strawberry CR isolate P414, two homologues, identical to each other (*PcAvh320*) were predicted adjacent to each other in the genome and were expressed upon infection of strawberry roots. This example of effector duplication therefore supplies the raw materials for adaptive evolution of the gene into novel functions in *P. cactorum*. Further long-read sequencing of additional isolates is required to determine the extent of CNV in *P. cactorum* as it is clear that short-read sequencing fails to detect instances of gene duplication with the same fidelity as long-read sequencing.

*In planta* transcriptome sequencing of further isolates will be able to investigate both effector expression variation and the role for CNV's, alongside functional characterisation and genetic analysis of the basis of resistance to *Phytophthora* in both strawberry and apple. This is non-trivial in both of these crops due to the lack of appropriate methods to transiently induce expression, as infiltration techniques into leaves are challenging due to leaf properties and until extremely recently genome sequences of the octoploid strawberry were not available to aid with the characterisation of putative resistance genes (Edger et al., 2019).

As with other *Phytophthora* spp. genomes, the advent of long-read sequencing technology has improved the generation of genome assemblies (Adams et al., 2020; Shi et al., 2021). In our study, a single strawberry CR isolate, P414, was sequenced with long read technology and was found to possess a greater genome size, number of gene models and greater numbers of genes, including RxLR and CRN effectors, compared to the other strawberry CR isolates sequenced by Illumina short-read technology. However, all comparative analyses (phylogenetic, orthogroup, expansion/contraction, and variant calling) performed within this study focus on shared characters across 13 isolates, comprised of this single long-read assembly and 12 short-read assemblies, and are not impacted by the prediction of additional genomic content. Therefore, the identification of potential host-determinants is a conservative one and there is the possibility of additional host-adapted effectors to be discovered, as additional long-read data is generated.

## Conclusion

This study provides further evidence that *P. cactorum* should be regarded as a species complex and not a single species, as it comprises of distinct phylogenetic lineages that resolve groups of isolates with distinct effector profiles and displaying host-preference. Pathotype specific effector genes, such as homologues to *PsAvh32* (*PcAvh215*) and *PiAvr3a* (*PcAvh258*) may play roles in specialisation of *P. cactorum* to strawberry and apple, respectively. However, functional analysis is required to validate these genes as determinants of pathogenicity in their respective host.

Further questions that also remain unanswered are to what extent do the expression profiles of effectors in different isolates affect pathogenicity? This highlights the need for further RNAseq from multiple isolates, as well as knockouts to disentangle which effectors are differential for pathogenicity in strawberry and apple? This will help us understand what are the key processes that underpin variation in virulence in *P. cactorum*.

This study raises questions about the strategy for effector-informed breeding. We know that clonal lineages predominate in strawberry, as has often been described in other agricultural-associated pathosystems (Hessenauer et al., 2021). We have shown that there are highly expressed lineage-specific effectors within the clonal lineage of CR along with CNV in highly expressed effectors. We hypothesise that these are associated with increased virulence and potentially also pathogenicity itself. If these were then used to screen for and then deploy resistance against them, then the question arises as to what would the response be, if the pathogen were to adapt? It could be that there are many other effectors that could play a similar role, that are currently silent within the genome, or conversely that through silencing of these effectors, host resistance may be evaded, but at a fitness cost to the pathogen. However, this remains to be seen. Conversely, we see that many “core” effectors that are conserved across lineages on different hosts are present but not expressed, so these, while we may assume they are important from DNA sequence data alone, they are clearly dispensable for pathogenicity, indicating that the idea of core effectors needs to expand beyond simply an analysis of their presence within a genome. Therefore, we must find ways to understand how the network of effectors functions in any given host and whether there are critical effector combinations that if targeted by multiple resistance genes would lead to a durable resistance.

## Data Availability Statement

The datasets, including raw data, assemblies, and annotations, generated for this study are available on NCBI GenBank as part of BioProjects PRJNA383548 and PRJNA391273, accession numbers are shown in Table 1. The BioSample IDs for the RNAseq data are SAMN18192675-SAMN18192689.

## Author Contributions

RH, CN, and AA devised the study, conceived, and drafted the manuscript. CN performed the experimental work, with input from LL, HB, and ML. AA performed the bioinformatic analyses including genome assembly, annotation, orthology gene expression, and variant calling analyses with input from MS. All authors read and approved the submission.

## Conflict of Interest

The authors declare that the research was conducted in the absence of any commercial or financial relationships that could be construed as a potential conflict of interest.

## Supplementary Material

Supplementary Figure 1 Example experimental setup of *in vitro Fragaria* × *ananassa* plants. **(A)** Strawberry plants positioned in petri dishes, on top of agar, **(B)** aluminium foil casing and upright positioning of plates in the growth incubator.

Supplementary Figure 2 All *Phytophthora cactorum* isolates tested were able to cause disease in strawberry fruit. Percentage of symptomatic strawberry ‘Elsanta' fruit after artificial inoculation *Phytophthora cactorum* and *Phytophthora idaei* zoospores, from three separate experiments.

Supplementary Figure 3 Agarose gel electrophoresis of RT-PCR β-tubulin reactions on representative samples from inoculation time course experiment of *Phytophthora cactorum* isolate P414 on the ‘Emily' cultivar of *Fragaria* × *ananassa*. L: 100 bp DNA Ladder (New England Biolabs). 1: Mock inoculated ‘Emily.' 2: 6 h post inoculation (hpi). 3: 12 hpi. 4: 24 hpi. 5: 48 hpi. 6: 72 hpi. 7: 96 hpi. 8: 120 hpi. 9: gDNA from ‘Emily' (negative control). 10: gDNA from P414 mycelium (positive control). 11: dH_2_O (negative control).

Supplementary Table 1 Characteristics of primers used in this study.

Supplementary Table 2 Details of genes expanded and contracted in the *Phytophthora cactorum* strawberry crown rot and apple lineages.

Supplementary Table 3 Summary of identified *Phytophthora cactorum* Avirulence gene homologues.

Supplementary Table 4 *Phytophthora cactorum* isolate P414 annotation table and *in planta* RNAseq analysis from *Fragaria* × *ananassa*.

Supplementary Table 5 Summary of homologues to known avirulence RxLR genes in *Phytophthora cactorum*. The region of genes for comparison are detailed in column three, noted as amino acid (AA) sequence after signal peptide (SP).

## References

[B1] Adams T. M. Armitage A. D. Sobczyk M. K. Bates H. J. Tabima J. F. Kronmiller B. A.. (2020). Genomic investigation of the strawberry pathogen Phytophthora fragariae indicates pathogenicity is associated with transcriptional variation in three key races. Front. Microbiol. 11:490. 10.3389/fmicb.2020.00490 32351458 PMC7174552

[B2] Alexander B. Stewart A. (2001). Glasshouse screening for biological control agents of *Phytophthora cactorum* on apple (*Malus domestica*). N. Zeal. J. Crop Horticult. Sci. 29, 159–169. 10.1080/01140671.2001.9514174

[B3] Anderson R. G. Deb D. Fedkenheuer K. McDowell J. M. (2015). Recent progress in RXLR effector research. Mol. Plant Microbe Interact. 28, 1063–1072. 10.1094/mpmi-01-15-0022-cr 26125490

[B4] Armitage A. D. Lysøe E. Nellist C. F. Lewis L. A. Cano L. M. Harrison R. J.. (2018). Bioinformatic characterisation of the effector repertoire of the strawberry pathogen Phytophthora cactorum. PLoS ONE 13:e0202305. 10.1371/journal.pone.0202305 30278048 PMC6168125

[B5] Armstrong M. R. Whisson S. C. Pritchard L. Bos J. I. B. Venter E. Avrova A. O.. (2005). An ancestral oomycete locus contains late blight avirulence gene Avr3a, encoding a protein that is recognized in the host cytoplasm. Proc. Natl. Acad. Sci. U.S.A. 7766–7771. 10.1073/pnas.0500113102 15894622 PMC1140420

[B6] Aronesty E. (2013). Comparison of sequencing utility programs. Open Bioinform. J. 7, 1–8 10.2174/1875036201307010001

[B7] Auwera G. A. V. Carneiro M. O. Hartl C. Poplin R. Angel G. Levy-Moonshine A.. (2013). From FastQ data to high confidence variant calls: the genome analysis toolkit best practices pipeline. Curr. Protoc. Bioinform. 43, 1–33. 10.1002/0471250953.bi1110s43 25431634 PMC4243306

[B8] Ball T. B. Plummer F. A. HayGlass K. T. (2003). Improved mRNA quantitation in LightCycler RT-PCR. Int. Arch. Allergy Immunol. 130, 82–86. 10.1159/000068372 12576739

[B9] Bankevich A. Nurk S. Antipov D. Gurevich A. A. Dvorkin M. Kulikov A. S.. (2012). SPAdes: a new genome assembly algorithm and its applications to single-cell sequencing. J. Comput. Biol. 19, 455–477. 10.1089/cmb.2012.0021 22506599 PMC3342519

[B10] Barboza G. E. Carrizo García C. Leiva González S. Scaldaferro M. Reyes X. (2019). Four new species of capsicum (*Solanaceae*) from the tropical andes and an update on the phylogeny of the genus. PLoS ONE 14:e0209792. 10.1371/journal.pone.0209792 30650102 PMC6334993

[B11] Barton N. (2010). Understanding adaptation in large populations. PLoS Genetics 6:e1000987. 10.1371/journal.pgen.1000987 20585547 PMC2887463

[B12] Bhat R. G. Colowit P. M. Tai T. H. Aradhya M. K. Browne G. T. (2006). Genetic and pathogenic variation in phytophthora cactorum affecting fruit and nut crops in California. Plant Dis. 90, 161–169. 10.1094/pd-90-0161 30786407

[B13] Boyer L. R. Feng W. Gulbis N. Hajdu K. Harrison R. J. Jeffries P.. (2016). The use of arbuscular mycorrhizal fungi to improve strawberry production in coir substrate. Front. Plant Sci. 7, 83–89. 10.3389/fpls.2016.01237 27594859 PMC4991251

[B14] Capella-Gutiérrez S. Silla-Martínez J. M. Gabaldón T. (2009). trimAl: a tool for automated alignment trimming in large-scale phylogenetic analyses. Bioinformatics 25, 1972–1973. 10.1093/bioinformatics/btp348 19505945 PMC2712344

[B15] Champouret N. Bouwmeester K. Rietman H. Lee T. Maliepaard C. Heupink A.. (2009). Phytophthora infestans isolates lacking class I ipiO variants are virulent on Rpi-blb1 potato. Mol. Plant Microbe Interact. 22, 1535–1545. 10.1094/MPMI-22-12-1535 19888819

[B16] Chen H. Shu H. Wang L. Zhang F. Li X. Ochola S. O.. (2018). Phytophthora methylomes are modulated by 6mA methyltransferases and associated with adaptive genome regions. Genome Biol. 19:181. 10.1186/s13059-018-1564-4 30382931 PMC6211444

[B17] Chepsergon J. Motaung T. E. Bellieny-Rabelo D. Moleleki L. N. (2020). Organize, don't agonize: strategic success of phytophthora species. Microorganisms 8:917. 10.3390/microorganisms8060917 32560346 PMC7355776

[B18] Cingolani P. Platts A. Wang L. L. Coon M. Nguyen T. Wang L.. (2012). A program for annotating and predicting the effects of single nucleotide polymorphisms, SnpEff: SNPs in the genome of Drosophila melanogaster strain w1118; iso-2; iso-3. Fly 6, 80–92. 10.4161/fly.19695 22728672 PMC3679285

[B19] Danecek P. Auton A. Abecasis G. Albers C. A. Banks E. DePristo M. A.. (2011). The variant call format and VCFtools. Bioinformatics 27, 2156–2158. 10.1093/bioinformatics/btr330 21653522 PMC3137218

[B20] DePristo M. A. Banks E. Poplin R. Garimella K. V. Maguire J. R. Hartl C.. (2011). A framework for variation discovery and genotyping using next-generation DNA sequencing data. Nat. Genet. 43, 491–498. 10.1038/ng.806 21478889 PMC3083463

[B21] Derevnina L. Contreras M. P. Adachi H. Cruces A. V. Xie R. Sklenar J.. (2021). Plant pathogens convergently evolved to counteract redundant nodes of an NLR immune receptor network. bioRxiv. 1–15. 10.1101/2021.02.03.429184 PMC8412950 34424903

[B22] Deutschmann V. F. (1954). Eine wurzelfäule an erdbeeren, hervorgerufen durch *Phytophthora cactorum* (Leb. et Cohn) Schröt. Nachr. Deutsch. Pflanzenschutzd. 6, 7–9

[B23] Dobin A. Davis C. A. Schlesinger F. Drenkow J. Zaleski C. Jha S.. (2013). STAR: ultrafast universal RNA-seq aligner. Bioinformatics 29, 15–21 10.1093/bioinformatics/bts635 23104886 PMC3530905

[B24] Edger P. P. Poorten T. J. VanBuren R. Hardigan M. A. Colle C. McKain M. R.. (2019). Origin and evolution of the octoploid strawberry genome. Nat. Genet. 51, 541–547. 10.1038/s41588-019-0356-4 30804557 PMC6882729

[B25] Ellis M. A. Grove G. G. (1983). Leather rot in ohio strawberries. Plant Dis. 67:549.

[B26] Erwin D. C. Ribeiro O. K. (1996). Phytophthora Diseases Worldwide. St. Paul, MN: American Phytopathological Society Press.

[B27] Gilroy E. M. Taylor R. M. Hein I. Boevink P. Sadanandom A. Birch P. R. J. (2011). CMPG1-dependent cell death follows perception of diverse pathogen elicitors at the host plasma membrane and is suppressed by phytophthora infestans RXLR effector AVR3a. New Phytol. 190, 653–666. 10.1111/j.1469-8137.2011.03643.x 21348873

[B28] Grenville-Briggs L. J. Kushwaha S. K. Cleary M. R. Witzell J. Savenkov E. I. Whisson S. C.. (2017). Draft genome of the oomycete pathogen Phytophthora cactorum strain LV007 isolated from European beech (*Fagus sylvatica*). Genom. Data 12, 155–156. 10.1016/j.gdata.2017.05.010 28560165 PMC5435576

[B29] Gurevich A. Saveliev V. Vyahhi N. Tesler G. (2013). QUAST: quality assessment tool for genome assemblies. Bioinformatics 29, 1072–1075. 10.1093/bioinformatics/btt086 23422339 PMC3624806

[B30] Haas B. J. Kamoun S. Zody M. C. Jiang R. H. Y. Handsaker R. E. Cano L. M.. (2009). Genome sequence and analysis of the irish potato famine pathogen *Phytophthora infestans*. Nature 461, 393–398. 10.1038/nature08358 19741609

[B31] Hantula J. Lilja A. Nuorteva H. Parikka P. Werres S. (2000). Pathogenicity, morphology and genetic variation of *Phytophthora cactorum* from strawberry, apple, rhododendron, and silver birch. Mycol. Res. 104, 1062–1068. 10.1017/S0953756200002999

[B32] Hantula J. Lilja A. Parikka P. (1997). Genetic variation and host specificity of *Phytophthora cactorum* isolated in Europe. Mycol. Res. 101, 565–572.

[B33] Harris D. C. (1991). The phytophthora diseases of apple. J. Horticult. Sci. 66, 513–544. 10.1080/00221589.1991.11516181

[B34] Hessenauer P. Feau N. Gill U. Schweddinger B. Brar G. S. Hamelin R. C. (2021). Evolution and adaptation of forest and crop pathogens in the anthropocene. Phytopathology 111, 49–67. 10.1094/PHYTP-08-20-0358-FI 33200962

[B35] Kamoun S. West P. Jong A. J. Groot K. E. Vleeshouwers V. G. A. A. Govers F. (1997). A gene encoding a protein elicitor of phytophthora infestans is down-regulated during infection of potato. Mol. Plant Microbe Interact. 10, 13–20. 10.1094/mpmi.1997.10.1.13 9002268

[B36] Katoh K. Standley D. M. (2013). MAFFT multiple sequence alignment software version 7: improvements in performance and usability. Mol. Biol. Evolut. 30, 772–780. 10.1093/molbev/mst010 23329690 PMC3603318

[B37] Koren S. Walenz B. P. Berlin K. Miller J. R. Bergman N. H. Phillippy A. M. (2017). Canu: scalable and accurate longread assembly via adaptive k-mer weighting and repeating. Genome Res. 27, 722–736. 10.1101/gr.215087.116 28298431 PMC5411767

[B38] Langmead B. Salzberg S. L. (2012). Fast gapped-read alignment with Bowtie 2. Nat. Methods 9, 357–359. 10.1038/nmeth.1923 22388286 PMC3322381

[B39] Lee H. Kim S. Oh S. Yeom S. Kim S. Kim M.. (2014). Multiple recognition of RXLR effectors is associated with nonhost resistance of pepper against *Phytophthora infestans*. New Phytol. 203, 926–938. 10.1111/nph.12861 24889686 PMC4143959

[B40] Lee H.-A. Lee H.-Y. Seo E. Lee J. Kim S.-B. Oh S.. (2017). Current understandings of plant nonhost resistance. Mol. Plant Microbe Interact. 30, 5–15. 10.1094/mpmi-10-16-0213-cr 27925500

[B41] Lilja A. Karjalainen R. Parikka P. Kammiovirta K. Nuorteva H. (1998). Pathogenicity and genetic variation of Phytophthora cactorum from silver birch and strawberry. Euro. J. Plant Pathol. 104, 529–535. 10.1023/A:1008644804415

[B42] Lin X. Song T. Fairhead S. Witek K. Jouet A. Jupe F.. (2020). Identification of Avramr1 from *Phytophthora infestans* using long read and cDNA pathogen-enrichment sequencing (PenSeq). Mol. Plant Pathol. 21, 1502–1512. 10.1111/mpp.12987 32935441 PMC7548994

[B43] Lin X. Witek K. Witek A. McLellan H. Nellist C. F. Armitage A. D.. (2019). The recognition of conserved RxLR effectors of *Phytophthora species* might help to defeat multiple oomycete diseases. Mol. Plant Microbe Interact. 32, S11–S1212. 10.1094/mpmi-32-10-s1.1 31573826

[B44] Liu K. Linder C. R. Warnow T. (2011). RAxML and FastTree: comparing two methods for large-scale maximum likelihood phylogeny estimation. PLoS ONE 6:e27731. 10.1371/journal.pone.0027731 22132132 PMC3221724

[B45] Longmuir A. L. Beech P. L. Richardson M. F. (2018). Draft genomes of two Australian strains of the plant pathogen, *Phytophthora cinnamomi*. F1000Research 6, 1972. 10.12688/f1000research.12867.1 29188023 PMC5698912

[B46] Love M. I. Huber W. Anders S. (2014). Moderated estimation of fold change and dispersion for RNA-seq data with DESeq2. Genome Biol. 15, 1–21. 10.1186/s13059-014-0550-8 25516281 PMC4302049

[B47] Luberti M. Litthauer S. Dunwell J. M. Fernández Fernández F. Nellist C. F. (2021). Response of apple (*Malus domestica*) accessions to UK *Phytophthora cactorum* isolates in cut-shoot tests. Acta Horticult. 1307, 369–374. 10.17660/ActaHortic.2021.1307.56

[B48] McKenna A. Hanna M. Banks E. Sivachenko A. Cibulskis K. Kernytsky A.. (2010). The genome analysis toolkit: a mapreduce framework for analyzing next-generation DNA sequencing data. Genome Res. 20, 1297–1303. 10.1101/gr.107524.110 20644199 PMC2928508

[B49] Naveed Z. A. Bibi S. Ali G. S. (2019). The phytophthora RXLR effector Avrblb2 modulates plant immunity by interfering with Ca2+ signaling pathway. Front. Plant Sci. 10:374. 10.3389/fpls.2019.00374 30984224 PMC6447682

[B50] Nellist C. F. Vickerstaff R. J. Sobczyk M. K. Marina-Montes C. Wilson F. M. Simpson D. W.. (2019). Quantitative trait loci controlling Phytophthora cactorum resistance in the cultivated octoploid strawberry (fragaria x ananassa). Horticult. Res. 6:60. 10.1038/s41438-019-0136-4 31069084 PMC6491645

[B51] Oh S. K. Young C. Lee M. Oliva R. Bozkurt T. O. Cano L. M.. (2009). In planta expression screens of phytophthora infestans RXLR effectors reveal diverse phenotypes, including activation of the solanum bulbocastanum disease resistance protein Rpi-blb2. Plant Cell 21, 2928–2947. 10.1105/tpc.109.068247 19794118 PMC2768934

[B52] Orsomando G. Lorenzi M. Raffaelli N. Rizza M. D. Mezzetti B. Ruggieri S. (2001). Phytotoxic protein PcF, purification, characterization, and cDNA sequencing of a novel hydroxyproline-containing factor secreted by the strawberry pathogen *Phytophthora cactorum*. J. Biol. Chem. 276, 21578–21584. 10.1074/jbc.m101377200 11262411

[B53] Patro R. Duggal G. Love M. I. Kingsford C. (2017). Salmon provides fast and bias-aware quantification of transcript expression. Nat. Methods 14, 417–419. 10.1038/nmeth.4197 28263959 PMC5600148

[B54] Pel M. A. (2010). Mapping, isolation and characterization of genes responsible for late blight resistance in potato (Ph.D. thesis), Wageningen, UR, 210. Available online at: https://edepot.wur.nl/138132

[B55] Pfaffl M. W. (2001). A new mathematical model for relative quantification in real-time RT–PCR. Nucleic Acids Res. 29:e45. 10.1093/nar/29.9.e45 11328886 PMC55695

[B56] Pfeifer B. Wittelsbürger U. Ramos-Onsins S. E. Lercher M. J. (2014). PopGenome: an efficient swiss army knife for population genomic analyses in R. Mol. Biol. Evolut. 31, 1929–1936. 10.1093/molbev/msu136 24739305 PMC4069620

[B57] Poppel P. M. J. A. Guo J. Vondervoort P. J. I. Jung M. W. M. Birch P. R. J. Whisson S. C.. (2008). The Phytophthora infestans avirulence gene Avr4 encodes an RXLR-dEER effector. Mol. Plant Microbe Interact. 21, 1460–1470. 10.1094/MPMI-21-11-1460 18842095

[B58] Qutob D. Tedman-Jones J. Dong S. Kuflu K. Pham H. Wang Y.. (2009). Copy number variation and transcriptional polymorphisms of phytophthora sojae RXLR effector genes Avr1a and Avr3a. PLoS ONE 4:e5066. 10.1371/journal.pone.0005066 19343173 PMC2661136

[B59] R Core Team (2019). R: A Language and Environment for Statistical Computing. Available online at: https://www.R-project.org/

[B60] Raj A. Stephens M. Pritchard J. K. (2014). fastSTRUCTURE: variational inference of population structure in large SNP data sets. Genetics 197, 573–589. 10.1534/genetics.114.164350 24700103 PMC4063916

[B61] Rietman H. Bijsterbosch G. Cano L. M. Lee H.-R. Vossen J. H. Jacobsen E.. (2012). Qualitative and quantitative late blight resistance in the potato cultivar sarpo mira is determined by the perception of five distinct RXLR effectors. Mol. Plant Microbe Interact. 25, 910–919. 10.1094/MPMI-01-12-0010-R 22414442

[B62] Rose D. H. (1924). Leather rot of strawberries. J. Agricult. Sci. 28, 357–376.

[B63] Rumold C. U. Aldenderfer M. S. (2016). Late archaic-early formative period microbotanical evidence for potato at jiskairumoko in the titicaca basin of southern Peru. Proc. Natl. Acad. Sci. U.S.A. 113, 13672–13677. 10.1073/pnas.1604265113 27849582 PMC5137686

[B64] Särkinen T. Bohs L. Olmstead R. G. Knapp S. (2013). A phylogenetic framework for evolutionary study of the nightshades (*Solanaceae*): a dated 1000-tip tree. BMC Evolut. Biol. 13:214. 10.1186/1471-2148-13-214 24283922 PMC3850475

[B65] Schmieder R. Edwards R. (2011). Quality control and preprocessing of metagenomic datasets. Bioinformatics 27, 863–864. 10.1093/bioinformatics/btr026 21278185 PMC3051327

[B66] Schulze-Lefert P. Panstruga R. (2011). A molecular evolutionary concept connecting nonhost resistance, pathogen host range, and pathogen speciation. Trends Plant Sci. 16, 117–125. 10.1016/j.tplants.2011.01.001 21317020

[B67] Seemüller E. Schmidle A. (1979). Einfluβ der herkunft von phytophthora cactorum-isolaten auf ihre virulenz an apfelrinde, erdbeerrhizomen und erdbeerfrüchten. Phytopathology 94, 218–225. 10.1111/j.1439-0434.1979.tb01553.x

[B68] Shi J. Ye W. Ma D. Yin J. Zhang Z. Wang Y.. (2021). Improved whole genome sequence of Phytophthora capsici generated by long-read sequencing. Mol. Plant Microbe Interact. 10.1094/mpmi-12-20-0356-a. [Epub ahead of print]. 33720746

[B69] Shulaev V. Sargent D. J. Crowhurst R. N. Mockler T. C. Folkerts O. Delcher A. L.. (2011). The genome of woodland strawberry (*Fragaria vesca*). Nat. Genet. 43, 109–116. 10.1038/ng.740 21186353 PMC3326587

[B70] Simão F. A. Waterhouse R. M. Ioannidis P. Kriventseva E. V. Zdobnov E. M. (2015). BUSCO: assessing genome assembly and annotation completeness with single-copy orthologs. Bioinformatics 31, 3210–3212. 10.1093/bioinformatics/btv351 26059717

[B71] Stam R. Jupe J. Howden A. J. M. Morris J. A. Boevink P. C. Hedley P. E.. (2013). Identification and characterisation CRN effectors in phytophthora capsici shows modularity and functional diversity. PLoS ONE 8:e59517. 10.1371/journal.pone.0059517.s005 23536880 PMC3607596

[B72] Stam R. Mantelin S. McLellan H. Thilliez G. (2014). The role of effectors in nonhost resistance to filamentous plant pathogens. Front. Plant Sci. 5:582. 10.3389/fpls.2014.00582/abstract 25426123 PMC4224059

[B73] Stensvand A. Herrero M. L. Talg,ø V. (1999). Crown rot caused by *Phytophthora cactorum* in norwegian strawberry production. EPPO Bull. 29, 155–158

[B74] Stukenbrock E. H. Bataillon T. (2012). A population genomics perspective on the emergence and adaptation of new plant pathogens in agro-ecosystems. PLoS Pathog. 8, e1002893–e1002894. 10.1371/journal.ppat.1002893 23028308 PMC3460620

[B75] Sugimoto T. Kato M. Yoshida S. Matsumoto I. Kobayashi T. Kaga A.. (2012). Pathogenic diversity of phytophthora sojae and breeding strategies to develop phytophthora-resistant soybeans. Breed. Sci. 61, 511–522. 10.1270/jsbbs.61.511 23136490 PMC3406798

[B76] Taylor A. Vágány V. Jackson A. C. Harrison R. J. Rainoni A. Clarkson J. P. (2016). Identification of pathogenicity-related genes in *Fusarium oxysporum* f. sp. cepae. Mol. Plant Pathol. 17, 1032–1047. 10.1111/mpp.12346 26609905 PMC4982077

[B77] Thomidis T. (2003). Variability in pathogenicity among greek isolates of *Phytophthora cactorum* to four peach rootstocks. Austr. J. Exp. Agricult. 43, 99–95. 10.1071/ea01203

[B78] Torto T. A. Li S. Styer A. Huitema E. Testa A. Gow N. A. R.. (2003). EST mining and functional expression assays identify extracellular effector proteins from the plant pathogen phytophthora. Genome Res. 13, 1675–1685. 10.1101/gr.910003 12840044 PMC403741

[B79] Wala J. A. Bandopadhayay P. Greenwald N. F. O'Rourke R. Sharpe T. Stewart C.. (2018). SvABA: genome-wide detection of structural variants and indels by local assembly. Genome Res. 28, 581–591. 10.1101/gr.221028.117 29535149 PMC5880247

[B80] Walker B. J. Abeel T. Shea T. Priest M. Abouelliel A. Sakthikumer S.. (2014). Pilon: an integrated tool for comprehensive microbial variant detection and genome assembly improvement. PLoS ONE 9:e112963. 10.1371/journal.pone.0112963 25409509 PMC4237348

[B81] Wang L. Chen H. Li J. Shu H. Zhang X. Wang Y.. (2019). Effector gene silencing mediated by histone methylation underpins host adaptation in an oomycete plant pathogen. Nucleic Acids Res. 313, 1261–1210. 10.1093/nar/gkz1160 31819959 PMC7039004

[B82] Wang Q. Han C. Ferreira A. O. Yu X. Ye W. Tripathy S.. (2011). Transcriptional programming and functional interactions within the *Phytophthora sojae* RXLR effector repertoire. Plant Cell 23, 2064–2086. 10.1105/tpc.111.086082 21653195 PMC3160037

[B83] Wang W. Jiao F. (2019). Effectors of Phytophthora pathogens are powerful weapons for manipulating host immunity. Planta 250, 413–425. 10.1007/s00425-019-03219-x 31243548

[B84] Wang Y. Wang Y. (2018a). Phytophthora sojae effectors orchestrate warfare with host immunity. Curr. Opin. Microbiol. 46, 7–13. 10.1016/j.mib.2018.01.008 29454192

[B85] Wang Y. Wang Y. (2018b). Trick or treat: microbial pathogens evolved apoplastic effectors modulating plant susceptibility to infection. Mol. Plant Microbe Interact. 31, 6–12. 10.1094/mpmi-07-17-0177-fi 29090656

[B86] Waterhouse R. M. Seppey M. Simão F. A. Manni M. Ioannidis P. Klioutchnikov G.. (2017). BUSCO applications from quality assessments to gene prediction and phylogenomics. Mol. Biol. Evolut. 35, 543–548. 10.1093/molbev/msx319 29220515 PMC5850278

[B87] Wawra S. Belmonte R. Löbach L. Saraiva M. Willems A. West P. (2012). Secretion, delivery and function of oomycete effector proteins. Curr. Opin. Microbiol. 15, 685–691. 10.1016/j.mib.2012.10.008 23177095

[B88] Wedgwood E. Berrie A. Passey T. Hall A. Xu X. (2020). Improving Integrated Disease Management in Strawberry. Available online at: https://projectblue.blob.core.windows.net/media/Default/Horticulture/SF%20157_Report_Final_2020.pdf

[B89] Wei C. Kvitko B. H. Shimizu R. Crabill E. Alfano J. R. Lin N.. (2007).A Pseudomonas syringae pv. tomato DC3000 mutant lacking the type III effector HopQ1-1 is able to cause disease in the model plant Nicotiana benthamiana. Plant J. 51, 32–46. 10.1111/j.1365-313x.2007.03126.x 17559511

[B90] Wilcox W. F. Scott P. H. Hamm P. B. Kennedy D. M. Duncan J. M. Brasier C. M.. (1993). Identity of a *Phytophthora species* attacking raspberry in Europe and North America. Mycol. Res. 97, 817–831. 10.1016/s0953-7562(09)81157-x

[B91] Win J. Chaparro-Garcia A. Belhaj K. Saunders D. G. O. Yoshida K. Dong S.. (2012). Effector biology of plant-associated organisms: concepts and perspectives. Cold Spring Harbor Symp. Quant. Biol. 77, 235–247. 10.1101/sqb.2012.77.015933 23223409

[B92] Yan H.-Z. Liou R.-F. (2006). Selection of internal control genes for real-time quantitative RT-PCR assays in the oomycete plant pathogen *Phytophthora parasitica*. Fungus Genet. Biol. 43, 430–438. 10.1016/j.fgb.2006.01.010 16531084

[B93] Yang M. Duan S. Mei X. Huang H. Chen W. Liu Y.. (2018). The *Phytophthora cactorum* genome provides insights into the adaptation to host defense compounds and fungicides. Sci. Rep. 8:6534. 10.1038/s41598-018-24939-2 29695739 PMC5916904

[B94] Yang X. Tyler B. M. Hong C. (2017). An expanded phylogeny for the genus phytophthora. IMA Fungus 8, 355–384. 10.5598/imafungus.2017.08.02.09 29242780 PMC5729717

[B95] Yu D. Tang H. Zhang Y. Du Z. Yu H. Chen Q. (2012). Comparison and improvement of different methods of RNA isolation from strawberry (Fragria x ananassa). J. Agricult. Sci. 4, 51–56. 10.5539/jas.v4n7p51

[B96] Yu G. Yu G. Smith D. K. Smith D. K. Zhu H. Zhu H.. (2016). ggtree: an r package for visualization and annotation of phylogenetic trees with their covariates and other associated data. Methods Ecol. Evolut. 8, 28–36. 10.1111/2041-210x.12628

[B97] Zhang C. Rabiee M. Sayyari E. Mirarab S. (2018). ASTRAL-III: polynomial time species tree reconstruction from partially resolved gene trees. BMC Bioinform. 19:153. 10.1186/s12859-018-2129-y 29745866 PMC5998893

[B98] Zhang S.-D. Jin J.-J. Chen S.-Y. Chase M. W. Soltis D. E. Li H.-T.. (2017). Diversification of rosaceae since the late cretaceous based on plastid phylogenomics. New Phytol. 214, 1355–1367. 10.1111/nph.14461 28186635

