# Supplementary material for: Comparative Analysis of Host-Associated Variation in Phytophthora cactorum
Source: Front Microbiol. 2021 Jul 2;12:679936. doi: 10.3389/fmicb.2021.679936 (PMC8285097; doi:10.3389/fmicb.2021.679936)
Supplement: Supplementary file 1 [file Data_Sheet_1.PDF]

SUPPLEMENTARY MATERIALS AND METHODS

Gene and open reading frame prediction

Gene prediction was performed on the softmasked *P. cactorum* genomes using BRAKER1 v.2 (Hoff et al., 2016), a pipeline for automated training and gene prediction of AUGUSTUS v3.1 (Stanke and Morgenstern, 2005). Evidence for gene models were produced using RNAseq data generated as part of this study (discussed below) and aligned to the *P. cactorum* assembly using STAR v2.5.3a (Dobin et al., 2013). Additional gene models were called in intergenic regions using CodingQuarry v.2.0 (Testa et al., 2015), which was run using the "pathogen" flag.

Gene models were also augmented with further effector candidates from open reading frames (ORFs) using the methods previously described in Armitage et al. (2018).

Functional annotation

Functional annotation of gene models was performed as described previously in Armitage et al. (2018). Putative CRNs were identified in predicted proteomes and translated ORFs by HMM (Hidden Markov Model) searches using LFLAK and DWL HMM models described in (Armitage et al., 2018). Putative RxLRs were identified following methods previously described by (Armitage et al., 2018). To confirm the absence of candidate genes in our genomes, a Basic Local Alignment Search Tool (BLAST) database was generated in Geneious Prime v2020.0 and used to confirm that genes were not present rather than not being in the respective annotation. PCR of gDNA was also used to confirm the presence/absence of the interesting RxLR candidates *PcAvh215* and *PcAvh258* in the representative *P. cactorum* isolates.

Phytophthora spp. zoospore production

The production of zoospores was followed from (Nellist et al., 2019). To summarise, 10 mm discs were excised from the edge of actively growing colonies, the plugs were covered with dilute compost extract in 90 mm triple-vented petri dishes (five per plate; Thermo Scientific) and were placed under constant light conditions at 20 ºC, for 48 hours, to stimulate sporangial development. After 48 hours, the diluted compost extract was poured off and replaced with a fresh solution. The plates were moved to a fridge (~4 ºC) and chilled for 45 min and then moved to the bench and left to warm up at room temperature for 45 min, to stimulate the release of zoospores. The suspension was then vacuum filtered through Whatman 113V Wet Strengthened 150 mm filter paper and the concentration of zoospores was determined using a haemocytometer and adjusted to 1×10^4, 2×10^4 or 5×10^3 zoospores per mL by diluting with dilute compost extract. The adjusted solution was kept on ice until ready to be used to inoculate plants/unripe fruit.

Pathogenicity tests on strawberry crowns

'Malling Opal' is an everbearer that is extremely susceptible to *P. cactorum*, bred by NIAB EMR (formally East Malling Research) and released in 2005. 'Elsanta' is a mid-season variety with reported susceptibility to crown rot. 'Fenella' is a mid-late season variety with good resistance to *P. cactorum*, bred by NIAB EMR and released in 2009.

Mother stock plants of the three cultivars were maintained in 1 L pots (Soparco) in polytunnels. Ten runners of each of the three cultivars were pinned down for each isolate into 9 cm diameter pots (Soparco) filled with peat-based compost. The clones were grown on for four and a half months and then placed in a 2 ºC coldstore for one week and then moved into a -2 ºC coldstore for at least two months. After two months the plants were removed from the coldstore and the dead leaves were removed. The plants were grown on for three weeks in a glasshouse compartment maintained at 20 ºC

during the day and 15 ºC at night, on a 16/8 hour, day/night light cycle, as described by Nellist et al. (2019).

The inoculation procedure for coldstored strawberry plants was performed as described in Nellist et al. (2019). Pathogenicity screens were performed under controlled conditions in glasshouse compartments, maintained at 20 ºC during the day and 15 ºC at night on a 16/8 day/night cycle for four weeks. Fifteen mm wounds were made at the base of a single petiole using a scalpel and ~5 mL of 1 x 10^4 zoospore suspension was sprayed across each wound and onto the compost. Plants were covered with clear plastic sheeting for 48 hours to maintain humidity. Scoring of symptoms was as described by Nellist et al. (2019) based upon a modified version from (Bell et al., 1997), where plants were scored on a scale of 1-8. Foliage was assessed visually for the presence of wilting symptoms, weekly over the four week period. If the plant died during the first, second, third or fourth week after inoculation, it was assigned a score of 8, 7, 6 or 5, respectively. The strawberry crowns were then cut open longitudinally and assessed on a scale of 1-5; 1 - healthy (0% infection), 2 - up to 25% infection, 3 - 26-50% infection, 4 - 51-75% infection, 5 - 76-100% infection. The data for the ten replicates were averaged and a mean crown rot disease score was used for further analysis. Statistical analyses were performed using R (v3.6.0, "Planting of a Tree" (Team, 2019). A one-way ANOVA was performed to analyse the difference between the pathogenicity of isolates cultured from strawberry on the three cultivars of strawberry.

Pathogenicity tests on excised apple shoots

Dormant first year growth apple shoots were collected from 'Cox' and 'Gala' in the Winter 2018. The processing of apple shoots was followed from Luberti et al. (2021). Briefly, the shoots were cut to 22 cm and surface sterilised for 15 minutes, by immersing them in a 10% bleach solution. The shoots were then rinsed three times with sterile distilled water and one centimetre was excised from each end. Both ends were sealed by dipping in molten paraffin wax. A wound was produced in the middle of each shoot using a 4 mm diameter cork borer and the outer bark was removed with a scalpel. Agar plugs of the same diameter, containing the leading edge of *P. cactorum* mycelium were placed mycelium-side down onto the wound to inoculate the shoots. Six shoots of each cultivar were inoculated per isolate. Mock inoculation of six shoots per cultivar were performed using sterile V8 agar plugs. The excised shoots were then transferred to boxes, damp paper towels were placed at the bottom of each box and the shoots were randomised by isolate and placed on raised racks made of aluminium foil. The boxes were sealed in clear plastic bags to maintain humidity and were placed in a controlled environment room, with a constant temperature of 22 ºC (±2 ºC) and a 16/8 hour, light/dark cycle for four weeks. Shoots were assessed for maximum lesion length at four weeks by removing the bark around the wound using a scalpel. A digital calliper was used to take measurements and the original wound size, 4 mm, was subtracted from each measurement. A one-way ANOVA was performed to analyse the difference between the pathogenicity of isolates cultured from apple and strawberry fruit on the two cultivars of apple.

RNA extraction from strawberry

Total RNA was extracted from the strawberry roots following a modified version of Yu et al. (2012), over two days. All solutions were made using RNase-free reagents and water. Mortar and pestles were decontaminated with RNAseZap^{TM} (Invitrogen/Thermo Fisher Scientific) and were baked for two hours at 230 ºC. Frozen root material and PVPP (0.01 g:0.1 g of frozen material) were weighed and ground with a mortar and pestle, in the presence of liquid nitrogen. The ground plant material was then split in half and transferred to 2x 700 µL of prewarmed (65 ºC) extraction buffer (3% CTAB, 100 mM Tris-HCL pH 8.0, 1.4 M NaCl, 20 mM EDTA, 5% PVP and RNase-free H_2O) with 10 µL of β-

mercaptoethanol. The remaining steps were then performed as previously described by (Yu et al., 2012) and the RNA was eluted into 65 µL DEPC-treated H_2O. The quantity and purity of the RNA were determined using the NanoDrop (ND-1000, Thermo Fisher Scientific) spectrophotometer. The Qubit 2 Fluorometer (Thermo Fisher Scientific) was also used to determine RNA quantity more accurately. RNA Integrity Number (RIN) was assessed by running the RNA on the Tapestation 4200 (Agilent Technologies). At least 1 µg of root RNA with a RIN score above 7 and with 260/280 and 260/230 ratios above 1.8 were sent to Novogene for sequencing. Strawberry root samples were sequenced to a depth of 50 million reads per sample.

REFERENCES

Armitage, A. D., Lysøe, E., Nellist, C. F., Lewis, L. A., Cano, L. M., Harrison, R. J., et al. (2018). Bioinformatic characterisation of the effector repertoire of the strawberry pathogen *Phytophthora cactorum*. *PLoS ONE* 13, e0202305-24. doi:10.1371/journal.pone.0202305.

Bell, J. A., Simpson, D. W., and Harris, D. C. (1997). Development of a method for screening Strawberry germplasm for resistance to *Phytophthora cactorum*. *Acta Horticulturae* 439, 175–179.

Dobin, A., Davis, C. A., Schlesinger, F., Drenkow, J., Zaleski, C., Jha, S., et al. (2013). STAR: ultrafast universal RNA-seq aligner. *Bioinformatics* 29, 15–21.

Hoff, K. J., Lange, S., Lomsadze, A., Borodovsky, M., and Stanke, M. (2016). BRAKER1: Unsupervised RNA-Seq-Based Genome Annotation with GeneMark-ET and AUGUSTUS. *Bioinformatics* 32, 767–769. doi:10.1093/bioinformatics/btv661.

Luberti, M., Litthauer, S., Dunwell, J.M., Fernández Fernández, F. and Nellist, C.F. (2021). Response of apple (Malus domestica) accessions to UK *Phytophthora cactorum* isolates in cut-shoot tests. *Acta Horticulturae* 1307, 369-374. doi:10.17660/ActaHortic.2021.1307.56.

Nellist, C. F., Vickerstaff, R. J., Sobczyk, M. K., Marina-Montes, C., Wilson, F. M., Simpson, D. W., et al. (2019). Quantitative trait loci controlling *Phytophthora cactorum* resistance in the cultivated octoploid strawberry (*Fragaria* x *ananassa*). *Horticulture Research* 6, 1–14. doi:10.1038/s41438-019-0136-4.

Stanke, M., and Morgenstern, B. (2005). AUGUSTUS: a web server for gene prediction in eukaryotes that allows user-defined constraints. *Nucleic Acids Research* 33, W465-7. doi:10.1093/nar/gki458.

Team, R. C. (2019). R: A language and environment for statistical computing. Available at: https://www.R-project.org/.

Testa, A. C., Hane, J. K., Ellwood, S. R., and Oliver, R. P. (2015). CodingQuarry: highly accurate hidden Markov model gene prediction in fungal genomes using RNA-seq transcripts. *BMC Genomics* 16, 170. doi:10.1186/s12864-015-1344-4.

Yu, D., Tang, H., Zhang, Y., Du, Z., Yu, H., and Chen, Q. (2012). Comparison and Improvement of Different Methods of RNA Isolation from Strawberry (*Fragria* x *ananassa*). *Journal of Agricultural Science* 4, 51–56. doi:10.5539/jas.v4n7p51.
