# Supplementary figures and images for: Comparative Analysis of Host-Associated Variation in Phytophthora cactorum
Source: Front Microbiol. 2021 Jul 2;12:679936. doi: 10.3389/fmicb.2021.679936 (PMC8285097; doi:10.3389/fmicb.2021.679936)

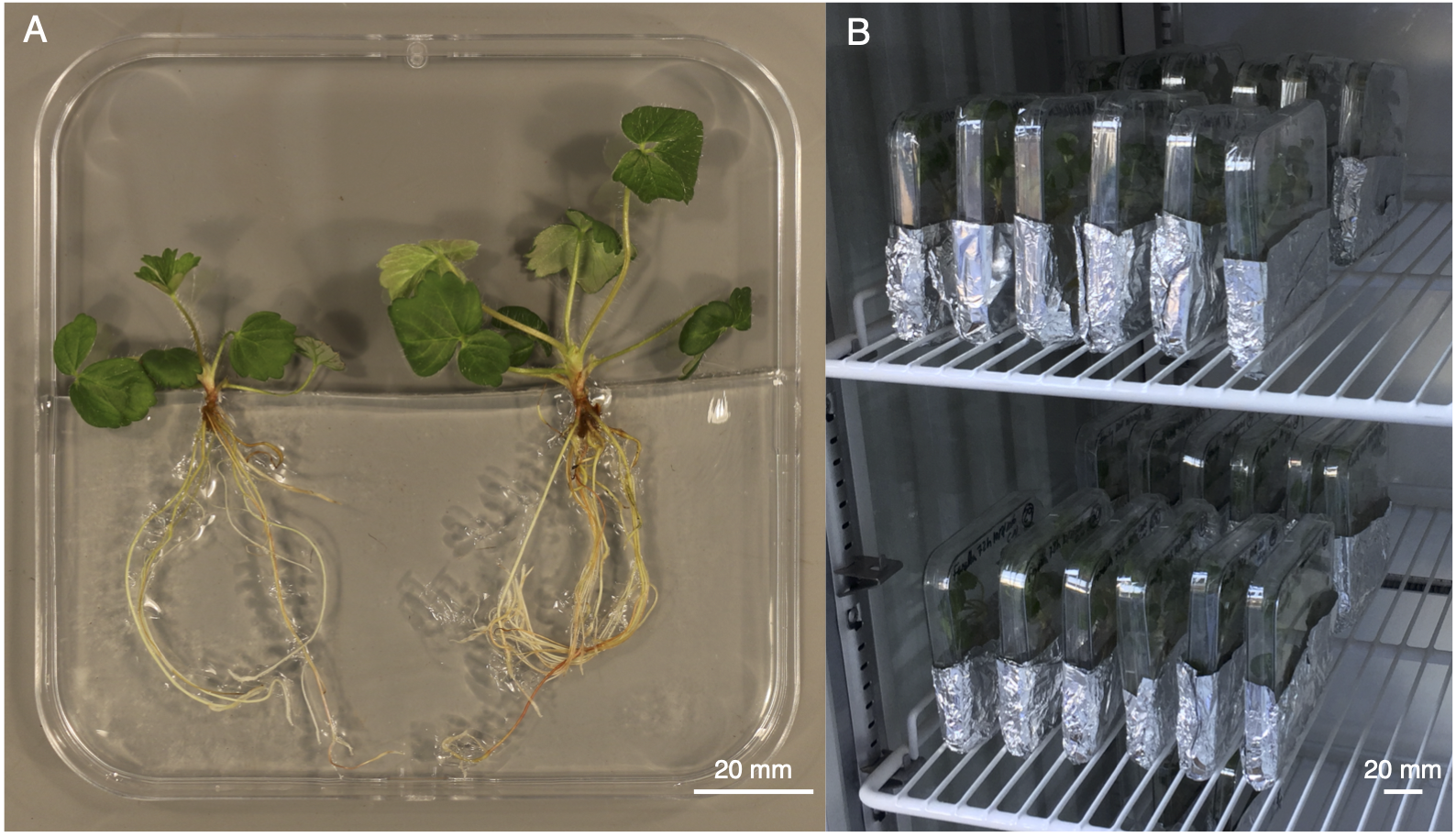

Supplement: Supplementary Figure 1 — Example experimental setup of in vitro Fragaria × ananassa plants. (A) Strawberry plants positioned in petri dishes, on top of agar, (B) aluminium foil casing and upright positioning of plates in the growth incubator. [file Image_1.TIFF]

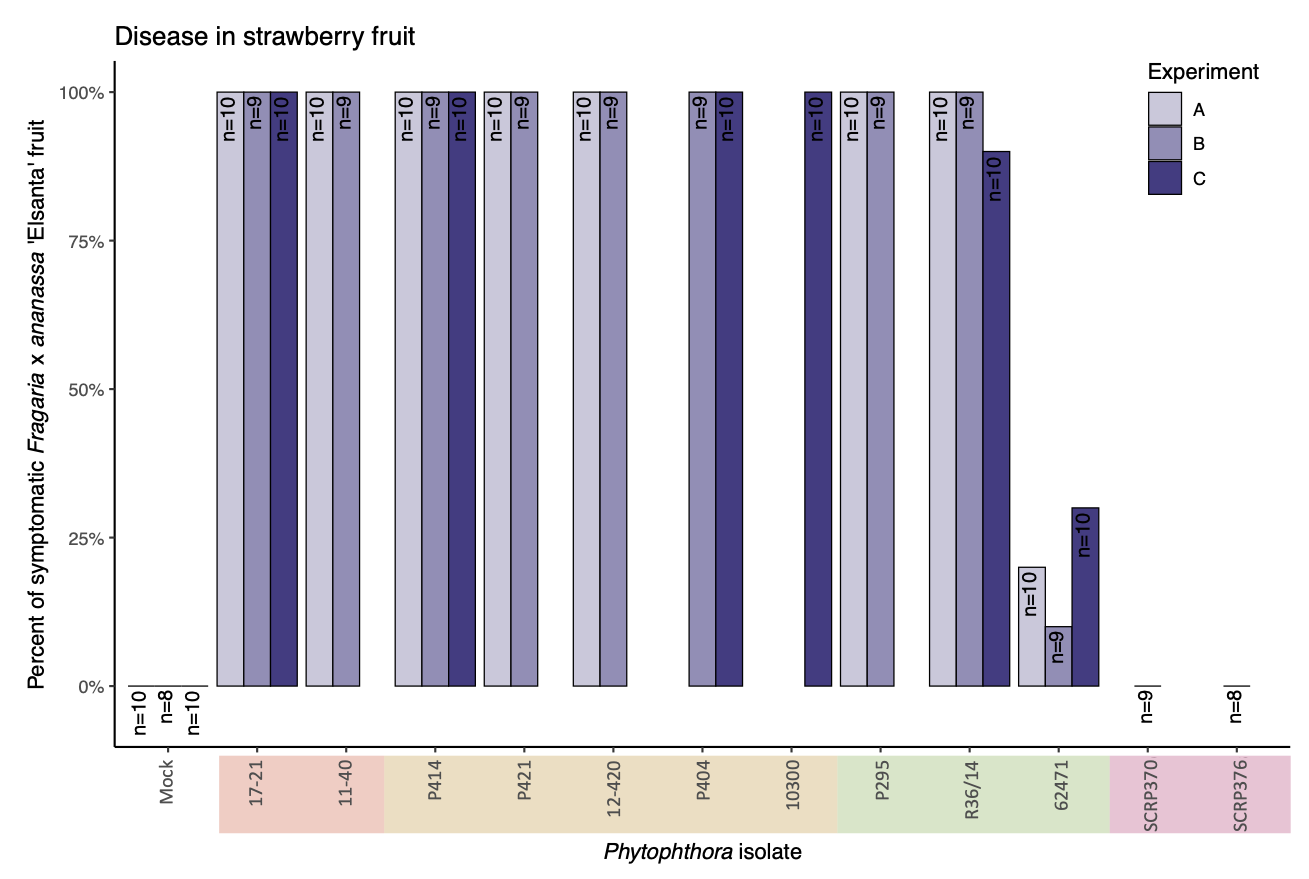

Supplement: Supplementary Figure 2 — All Phytophthora cactorum isolates tested were able to cause disease in strawberry fruit. Percentage of symptomatic strawberry ‘Elsanta' fruit after artificial inoculation Phytophthora cactorum and Phytophthora idaei zoospores, from three separate experiments. [file Image_2.TIFF]

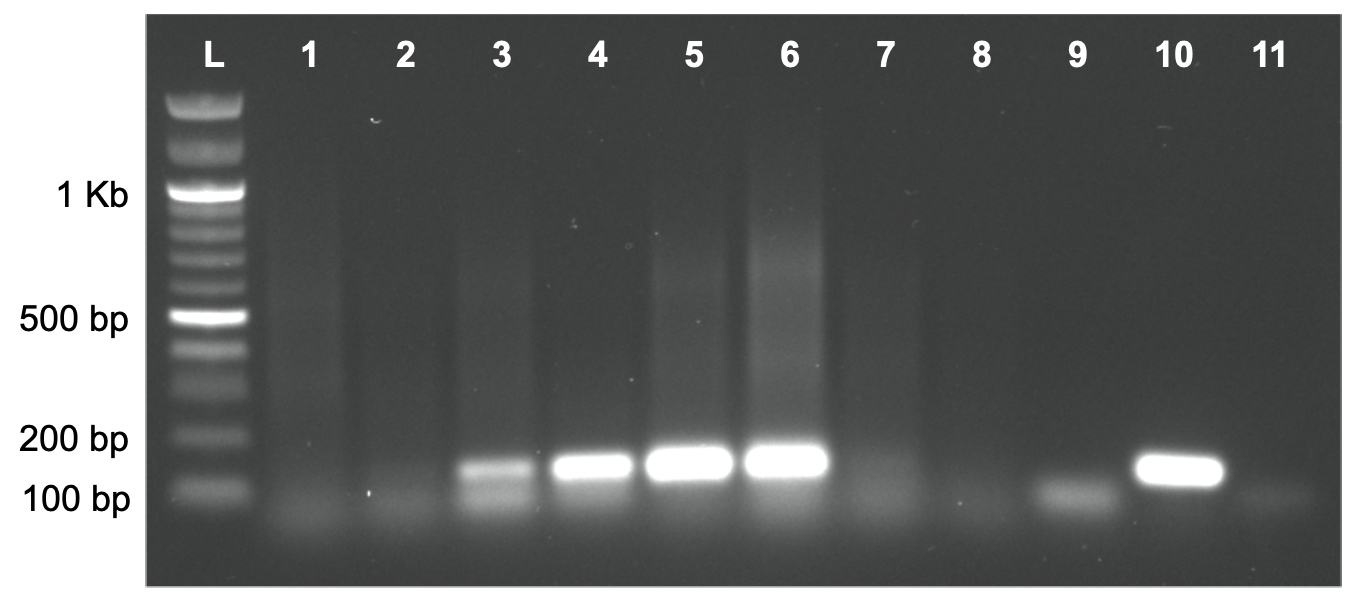

Supplement: Supplementary Figure 3 — Agarose gel electrophoresis of RT-PCR β-tubulin reactions on representative samples from inoculation time course experiment of Phytophthora cactorum isolate P414 on the ‘Emily' cultivar of Fragaria × ananassa. L: 100 bp DNA Ladder (New England Biolabs). 1: Mock inoculated ‘Emily.' 2: 6 h post inoculation (hpi). 3: 12 hpi. 4: 24 hpi. 5: 48 hpi. 6: 72 hpi. 7: 96 hpi. 8: 120 hpi. 9: gDNA from ‘Emily' (negative control). 10: gDNA from P414 mycelium (positive control). 11: dH2O (negative control). [file Image_3.TIFF]
