# Supplementary material for: Comparative Analysis of Host-Associated Variation in Phytophthora cactorum
Source: Front Microbiol. 2021 Jul 2;12:679936. doi: 10.3389/fmicb.2021.679936 (PMC8285097; doi:10.3389/fmicb.2021.679936)
Supplement: Supplementary Table 5 — Summary of homologues to known avirulence RxLR genes in Phytophthora cactorum. The region of genes for comparison are detailed in column three, noted as amino acid (AA) sequence after signal peptide (SP). [file Table_5.DOCX]

| **KNOWN  AVR** | **GENBANK ACC.** | **AA (AFTER SP)** | **PcAvh ID** | **P414 GENE ID**  **(Pcac1_)** | **P414 EXP.** | **R36/14 GENE ID**  **(PC123_)** | **R36/14 EXP.** | **17-21 GENE ID**  **(PC128_)** | **17-21 EXP.** | **% SIMILARITY** | **NOTES** |
| --- | --- | --- | --- | --- | --- | --- | --- | --- | --- | --- | --- |
| PiAvr1 | DS028168.1 | 23-209 | PcAvh210/1/2 | g15762 | Yes | g15192 | -- | g15282 | -- | 52.5 |  |
| PiAvr2 | XM_002902939.1 | 21-117 | -- | -- | -- | -- | -- | -- | -- | -- |  |
| PiAvr3a | AEH27535.1 | 22-147 | PcAvh258 | -- | -- | g19522 | Yes | -- | -- | 57.9 |  |
| PiAvr3b | XM_002998411.1 | 20-254 | PcAvh377/78/79/80/81/82 | g11509/g11873/ g11938 | No | g22203/g15619/ g6721 | -- | g10109/g18031/ g4369 | -- |  |  |
| PiAvr4 | EF672355.1 | 25-287 | PcAvh356/7 | g4103 | No | g3490 | -- | g15741 | -- | 52.6 | No start codon in R36/14 & 17-21, genes called wrong |
| PiAvrblb1 | EEY61733.1 | 22-152 | PcAvh394 | g3106 | Yes | g26318 | -- | g18559 | -- | 41.7 | Also known as ipiO |
| PiAvrblb2 | XM_002895872.1 | 23-100 | PcAvh320 | g16030/1 | Yes | g27333 | No | g26879 | Yes | 26.4 |  |
| PiAvrvnt1 | XM_002997111.1 | 24-154 | PcAvh428/9 | g7005 | No | g16330 | -- | g11784 | -- | 56.8 |  |
| PiAvrSmira1 | KX887490.1 | 24-238 | PcAvh266/7/8 | g15770 | Yes | g18579 | -- | g6728 | -- | 35.6 |  |
| PiAvr8  (PiAvrSmira2) | XM_002904498.1 | 21-244 | -- | g3290 | No | g17704 | -- | g16289 | -- | 50.0 | Not called as RxLR in P414, introns in R36/14 & 17-21 |
| PiAvramr1 | XM_002904507.1 | 22-287 | PcAvh335 | g9245 | Yes | g12607 | Yes | g9824 | Yes | 61.3 |  |
| PiAvramr3 | XM_002895186.1 | 23-339 | PcAvh092 | g26927 | No | g24106 | No | g11927 | No | 62.1 |  |
